# Endocrine system after 2 years of COVID-19 vaccines: A narrative review of the literature

**DOI:** 10.3389/fendo.2022.1027047

**Published:** 2022-11-10

**Authors:** Letizia Chiara Pezzaioli, Elisa Gatta, Francesca Bambini, Paolo Facondo, Maria Gava, Maria Cavadini, Caterina Buoso, Elena Di Lodovico, Mario Rotondi, Alberto Ferlin, Carlo Cappelli

**Affiliations:** ^1^ Department of Clinical and Experimental Sciences, Unit of Endocrinology and Metabolism, University of Brescia, Brescia, Italy; ^2^ Istituti Clinici Scientifici Maugeri IRCCS, Unit of Internal Medicine and Endocrinology, Laboratory for Endocrine Disruptors, Pavia, Italy; ^3^ Department of Internal Medicine and Therapeutics, University of Pavia, Pavia, Italy; ^4^ Department of Medicine, Unit of Andrology and Reproductive Medicine, University of Padova, Padova, Italy

**Keywords:** COVID-19, COVID-19 vaccines, endocrine system, SARS-CoV-2 vaccine, endocrine adverse effects

## Abstract

**Purpose:**

The purpose of this study was to describe the current knowledge on the potential endocrine adverse effects post-COVID-19 vaccines.

**Methods:**

A PubMed/MEDLINE, Web of Science, and Scopus research was performed. Case reports, case series, original studies, and reviews written in English and published online up to 31 July 2022 were selected and reviewed. The final reference list was defined based on the relevance of each paper to the scope of this review.

**Results:**

The available data showed that endocrine side effects are generally rare and with favorable outcome, being thyroid disorders the most common. Conversely, data on type 1 diabetes mellitus are rare; adrenal and pituitary events are even anecdotal. Finally, the available clinical studies suggest no impact on female reproductive system and on male and couple fertility.

**Conclusion:**

Overall, these data show that, after 2 years of COVID-19 vaccines, the endocrine system is not heavily threatened.

## Introduction

Since its appearance at the end of 2019, coronavirus disease 2019 (COVID-19) has globally affected over 580 million people, including over 6 million deaths reported to World Health Organization (WHO) (1).

While known to primarily affect the respiratory system, it has become evident that many relevant extrapulmonary manifestations contribute to the severity of COVID-19 (2), including cardiovascular, renal, gastrointestinal, urinary, and endocrine systems (3, 4). This marked that the variability of clinical manifestations has been attributed to the presence in all these tissues of angiotensin converting enzyme 2 (ACE2) receptor, which plays a pivotal role in COVID-19 pathogenesis (5).

Many vaccines have been developed to induce protection against severe COVID-19 manifestations, and, as of 9 August 2022, over 12 billion vaccine doses have been administered (1). WHO, as of 8 April 2022, confirmed the safety and effectiveness of the following vaccines: Moderna mRNA-1273, Pfzer/BioNTech BNT162b2, AstraZeneca/Oxford Vaxzevria ChAdOx1, Janssen Ad26.COV2, Sinopharm BIBP, Sinovac Coronavac, Covaxin BBV512, Convidecia AD5-nCOV, Covovax, and Nuvaxovid NVX-CoV2373 (6). Most of these vaccines contain messenger RNA (mRNA) or inactivate or vectorial virus that induces protective immune response. In detail, mRNA-based vaccines are encapsulated in lipid nanoparticles to transport nucleoside-modified mRNA encoding viral proteins to the host cell membrane; conversely, vaccines based on adenoviral or human vector are designed to invade cells without replication, whereas inactivated vaccines are chemically obtained and formulated with adjuvants (7).

The overall safety and efficacy of the available COVID-19 vaccines have been confirmed in multiple studies and metanalyses (8), even if the occurrence of a few cases of post-vaccination complications have been observed (9), including endocrine manifestations.

Besides the effects on hormonal structure (10) and long-term endocrine-metabolic complications (11), many different cases of endocrine adverse effects after COVID-19 vaccines have been reported, mainly as case reports/series. However, a comprehensive review is needed, especially because the endocrine system has been prone to many misconceptions regarding COVID-19 vaccines (3).

Therefore, we aimed to review the current findings on the effects of COVID-19 vaccines on endocrine system.

## Search strategy and selection criteria

A PubMed/MEDLINE, Web of Science, and Scopus research was performed, for free-text words and terms related to “Severe acute respiratory syndrome coronavirus 2 (SARS-CoV-2) vaccine”, “COVID-19 vaccine”, “SARS-CoV-2 vaccination”, “COVID-19 vaccination”, “SARS-CoV-2 immunization” variously combined with “endocrinopathies”, “endocrine system”, “thyroid”, “subacute thyroiditis”, “Graves’ disease”, “hypothyroidism”, “hyperthyroidism”, “adrenal”, “adrenal insufficiency”, “adrenal crisis”, “Addison disease”, “adrenal haemorrhage”, “pituitary gland”, “hypophysitis”, “hypopituitarism”, “pituitary apoplexy”, “type 1 diabetes”, “diabetic ketoacidosis”, “diabetes”, “ovary”, “amenorrhea”, “menstrual cycle”, “menstrual dysfunction”, “female fertility”, “male infertility”, “couple infertility”, “sperm”, “reproductive health”, “male hypogonadism”, and “sexual dysfunction”.

Case reports, case series, original studies, and reviews written in English and published online up to 31 July 2022 were selected and reviewed. The final reference list was defined based on the relevance of each paper to the scope of this review.

## Results

### Thyroid and COVID-19 vaccine

To date, 172 cases of thyroid involvement after COVID-19 vaccine have been reported as case reports/case series and are briefly summarized in **Table 1**. Subacute thyroiditis (SAT) is the most frequent finding, followed by new or recurrent Graves’ disease. Few clinical studies have been recently published, concerning the possible alterations of thyroid function and SAT characteristics, as shown in **Supplementary Table 2**.

**Table 1:** Summarized data from case reports regarding thyroid gland, adrenal involvement, pituitary function alteration, type 1 diabetes mellitus (T1DM) occurrence/worsening, and male. reproductive function after COVID-19 vaccine.

Adverse effect (AE)	Total of reported cases	Vaccine type			Sex (M/W)	Age (median)	Timing of onset (days)	Dose after which the AE occurred			Need for immediate treatment	Need for long term therapy	Deadly events
		mRNA vaccine	Inactivated virus	Viral vector				1^st^	2^nd^	≥3^rd^			
**Thyroid gland**	172	117	26	27									
*SAT*	98	58	25	13	24/74	41	10	48	43	5	85	15	0
*Graves’*	64	50	1	13	17/47	42.5	10	35	27	1	55	5	0
*Painless thyroiditis*	5	4	0	1	1/4	34	7	4	1	0	1	0	0
*Hypothyroidism*	2	2	0	0	1/1	61	18	0	1	1	2	2	0
*Other alterations*	3	3	0	0	1/2	64	4	2	0	1	2	1	0
**Adrenal gland**	16	1	0	15									
*Adrenal haemorrhage*	9	0	0	9	5/4	46	8	7	0	0	9	7	2
*Adrenal crisis*	6	1	0	5	3/3	69	1	5	1	0	6	6	0
*Pheochromocytoma*	1	0	0	1	1/0	63	1	1	0	0	1	1	0
**Pituitary gland**	8	2	0	2									
*Hypophysitis*	2	2	0	0	1/1	50	2	1	1	0	2	2	0
*SIADH*	1	1	0	0	1/0	79	10	0	1	0	1	1	0
*Pituitary apoplexy*	2	0	0	2	0/2	32.5	3	2	0	0	0	0	0
*Central diabetes insipidus*	1	1	0	0	0/1	37	7	0	1	0	1	1	0
*ACTH deficit*	2	2	0	0	2/0	39.5	5	1	1	0	2	2	0
**Type 1 diabetes mellitus**	21	17	2	2									
*New onset*	15	13	1	1	7/8	50	6	4	10	1	13	12	0
*Glycaemic alterations*	6	4	1	1	2/4	24.5	1	1	5	0	6	5	0
**Male reproductive system**	1	1	0	0									
*Sperm parameters*	1	1	0	0	1/0	43	NA	0	0	1	0	0	0

AE, adverse effects; T1DM, type 1 diabetes mellitus; SAT, subacute thyroiditis, SIADH, syndrome of inappropriate antidiuresis; ACTH, adrenocorticotropic hormone; NA, Not Available.

#### Subacute thyroiditis

A total of 98 cases of SAT after COVID-19 vaccine were retrieved, based on 46 articles, as summarized in **Table 1**. Complete data are available in **Supplementary Table S1**.

Median age of the 98 patients was 41 years (37–53), and 74 of them (75.5%) were women. In 14 patients (18.4%), a previous diagnosis of thyroid disease was reported, including primary hypothyroidism (18, 19), multinodular goiter (20), previous SAT (20–22) or Graves’ disease (21), multinodular goiter (20, 23), and papillary thyroid carcinoma (22).

Data concerning the type of vaccine were available for 96 patients. In detail, 58 patients (60.4%) received an mRNA vaccine (Pfizer/BioNTech BNT162b2 or Moderna mRNA-1273), 13 (13.5%) a viral vector vaccine (Vaxzevria ChAdOx1 or Convidecia AD5-nCOV), and 25 (26%) an inactivated virus (Sinovac Biotech CoronaVac or Bharat Biotech BBV152). In 48 cases (50%), SAT followed the first vaccine dose, in 43 cases (44.7%) the second one, and in five cases (5.2%) the third or subsequent doses. Patients developed SAT after a median of 10 days (4–11, 18–21) after the vaccine shot, independently from the type of vaccine administered. All the patients were symptomatic, being the most common symptoms anterior neck pain, fever, palpitations, fatigue, and weight loss. Concerning treatment, 39 patients (40.6%) were treated with corticosteroids (methylprednisolone, prednisone, or prednisolone), 31 (32.3%) with beta-blockers, 50 (52%) with nonsteroidal anti-inflammatory (NSAIDs), and only one (1%) with methimazole; nine patients (9.3%) did not receive any specific treatment.

Regarding follow-up, overt hypothyroidism was diagnosed in 14 cases and subclinical hypothyroidism was found in two patients; in nine cases, levothyroxine treatment was given. There was a recurrence of SAT after a subsequent vaccine dose in four cases (three after Pfizer/BioNtech BNT16b2 and one after Sinovac Biotech CoronaVac) (20, 24, 25).

Recently, four clinical studies have been published concerning SAT and COVID-19 vaccines, as shown in **Supplementary Table 2**.

In detail, Garcia et al. (12), in a large case/non-case study, analyzed 1,221,582 spontaneous cases of adverse reactions with mRNA and viral vector vaccines and found 162 cases of SAT, with a median time to onset of 10 days after any vaccine. They observed that SAT occurred more frequently after mRNA vaccines (reporting odds ratio of 3.58 and 3.44 for Pfizer/BioNtech BNT16b2 and Moderna mRNA-1273, respectively).

Bostan et al. (16) retrospectively examined 55 patients diagnosed with SAT, of whom 16 had previously undergone COVID-19 vaccine, with a median of 6.5 days before SAT development. They found no differences concerning the characteristics of vaccine.

Conversely, Topaloglu et al. (17) compared 23 cases of vaccine-related SAT with 62 cases of “classical” SAT; they found that SAT duration was longer in vaccinated patients compared with non-vaccinated ones.

Finally, Sendur et al. (26) performed a case-control study, including 14 patients with COVID-induced SAT, in order to assess the possible associations with specific haplotypes. With the limited sample size and the lack of many clinical information, they observed that Human Leukocyte Antigen (HLA)-B35 and HLA-C04 alleles were higher in patients with vaccine-induced SAT compared with healthy controls.

#### Graves’ disease

Concerning Graves’ disease, 29 articles were found and a total of 64 cases after COVID-19 vaccination were retrieved and are summarized in **Table 1**, whereas complete data are available in **Supplementary Table S1**.

The median age of the 64 patients was 42.5 years (35–53), and 47 of them (73.4%) were women.

First-onset Graves’ disease was diagnosed in 54 patients, including recently described cases (27, 28), whereas a relapse of a previously known and quiescent Graves’ disease was reported in 10 cases (14, 29–32), of whom five (14, 30, 31) developed an ophthalmopathy. Besides the patients with a previous diagnosis of Graves’ disease, further four patients had a known thyroid disease, including multinodular goiter (15), subclinical hypothyroidism under replacement therapy (33), Hashimoto’s thyroiditis (34), and an unspecified form of hyperthyroidism (31).

Of the 64 patients, 50 (78.1%) received an mRNA vaccine (Pfizer/BioNTech BNT162b2 or Moderna mRNA-1273), 13 (20.3%) received a viral vector vaccine (Vaxzevria ChAdOx1 or Convidecia AD5-nCOV or Jennsen), and only one (1.6%) received an inactivated virus vaccine (Sinovac Biotech CoronaVac). Median time between the vaccine shot (any type) and the symptoms’ development was 10 days (4–11, 18–23). Concerning the relationship between the dose of vaccine and Graves’ disease onset, 35 patients (54.7%) became symptomatic after the first dose, in 27 (42.2%) after the second dose, and in one case after the third dose. The most common symptoms and signs were anxiety, insomnia, irritability, diaphoresis, palpitation, tachycardia, hand tremors, and weight loss. Furthermore, five patients showed signs and symptoms of Graves’ ophthalmopathy (14, 30, 31).

Concerning treatment, Methimazole or Carbimazole, with or without beta blockers was prescribed to 51 patients (87.9%) and intravenous Teprotumumab infusions were given to four patients (6.9%) (14, 30, 31), whereas two patients (3.4%) (31, 35) did not receive any specific treatment.

Regarding follow-up, thyroid function returned to normal and symptoms improved in most cases; only four patients (23, 36–38) were still hyperthyroid and one (32) eventually required total thyroidectomy.

#### Other thyroid function alterations

Regarding thyroid function alteration after COVID-19 vaccine, 10 cases were retrieved, as shown in **Supplementary Table S1**, and briefly summarized in **Table 1**.

In detail, five patients (four women and one man, aged 29–59 years) developed a painless thyroiditis after receiving an mRNA vaccine or viral vector vaccine, with a median time to onset of symptoms of 7 days. In almost all the cases, symptoms were developed after the first dose and none of the patients required specific treatment. Thyroid function was spontaneously normalized in all cases.

Moreover, two cases of overt hypothyroidism, one case of autoimmune thyroiditis with normal thyroid function and one case of thyrotoxicosis, were reported, all occurring after mRNA vaccine administration (first dose in two cases and third in one).

Recently, four clinical studies have been published concerning thyroid function alteration related to COVID-19 vaccine, as shown in **Supplementary Table 2**. In detail, three of them (39–41) concerned the possible alteration of thyroid hormones and thyroid antibodies in healthy people receiving mRNA or inactivated vaccines. They showed little (or no) modification of thyroid stimulating hormone (TSH) and free thyroid hormones, which remained within normal ranges. As for the autoantibodies titre, only Lui et al. (40) found a slight increase of thyroid peroxidase antibodies (TPOAb) and thyroglobulin antibodies (TgAb).

Finally, Xiong et al. (42) evaluated, in a large retrospective cohort study, the risk of vaccine related adverse events in hypothyroid patients under levothyroxine treatment. They observed that, after two doses, both Pfizer/BioNtechBNT162b2 and Sinovac Biotech Coronavac were not associated with alteration of thyroid status.

### Adrenal gland and COVID-19 vaccine

Adrenal adverse effects of COVID-19 vaccination were reported for 16 patients, as briefly summarized in **Table 1**. Complete data are available in **Supplementary Table S1**.

Of note, nine of the 16 cases (43–50) concerned episodes of adrenal insufficiency due to adrenal haemorrhage. In detail, median age of these patients was 46 years, five of nine were men, and four had predisposing factors of thromboembolism, including smoke (45, 49) and obesity (49, 50). In all cases, patients had received viral vector vaccines, Vaxzevria ChAdOx1 in eight patients (43, 44, 46, 47) and Johnson and Johnson in one (45). Clinical manifestations developed 8 days (median time) after the first dose of vaccine and included systemic thromboembolic and hemorrhagic complications, in addition to the aforementioned adrenal hemorrhage. In two patients, clinical presentation was fatal (46, 47). A diagnosis of vaccine-induced immune thrombotic thrombocytopenia (VITT) was made in seven cases (43–45, 48–50), and anti-platelet factor 4 (PF-4) antibodies were detected in eight patients (43, 44, 47–50).

Moreover, Maguire et al. (51) and Markovic et al. (52) described collectively six cases of adrenal crisis or incipient crisis, occurred after the administration of Vaxzevria ChAdOx1 vaccine (five patients) (51) or Pfizer/BioNtechBNT162b2 (one patient) (52). All the patients had a previous diagnosis of adrenal insufficiency, under proper replacement therapy. Adverse reactions developed within 24h after vaccination and caused adrenal crises, and hospitalization was needed in four cases. All the adrenal crises were promptly resolved by increasing the replacement therapy regimen.

Finally, Haji et al. (53) described a case of pheochromocytoma multisystem crisis occurred within 24h after the first dose of the Johnson and Johnson COVID-19 vaccine in a 63-years-old man. His clinical presentation was so severe to determine respiratory distress, acute kidney injury, cardiogenic shock, and cardiomyopathy. Once respiratory and hemodynamic stability were obtained, the patient was successfully treated with adrenal mass resection.

### Pituitary gland and COVID-19 vaccine

Pituitary gland disfunctions after COVID-19 vaccine, including hypophysitis, pituitary apoplexy (PA), adrenocorticotropic hormone (ACTH) deficiency, syndrome of inappropriate antidiuresis (SIADH), and central diabetes insipidus were reported in eight cases, as briefly shown in **Table 1** (and extensively in **Supplementary Table S1**).

Regarding hypohphysitis, two cases were reported (54, 55), concerning a man and a woman; median age was 50 years; in both cases, symptoms of hypopituitarism occurred shortly after mRNA-based vaccines (one after first dose and one after second dose). Imaging findings and blood tests were consistent with hypophysitis, with central adrenal insufficiency and secondary hypothyroidism in one case (54) and central diabetes insipidus in the other one (55), both requiring long term hormonal replacement therapy.

Concerning PA, two cases (56, 57) were reported, both regarding young women (28–37 years), who showed intense headache 1–5 days after the first dose of Vaxzevria ChAdOx1 (56, 57). In both patients, haemorrhagic bleeding of the adenohypophysis was documented and in none of the cases any medical treatment was needed.

Isolated ACTH deficit was observed in two cases (58, 59), both occurring in young men (31–48 years) shortly after Pfizer/BioNtechBNT162b2 vaccine (first dose in one case and second dose in the other one). In one case (58), adrenal insufficiency due to ACTH deficit was the only clinical manifestation, whereas the patient described by Mizuno et al. (59) was diagnosed with concomitant neuroleptic malignant syndrome and ACTH deficit. Both patients were given adequate steroid replacement therapy with clinical improvement.

Finally, two more cases were reported, one concerning SIADH (60) occurred in a 79-year-old woman 10 days after the second dose of the Moderna mRNA-1273 and one regarding the onset of central diabetes insipidus (61) in a 37-year-old woman 7 days after the second dose of Pfizer/BioNtechBNT162b2 vaccine. In both cases, adequate treatment with desmopressin (61) or with intravenous crystalloid solutions, fluid restriction and oral urea (60) led to rapid improvement of the symptoms.

### Type 1 diabetes mellitus and COVID-19 vaccine

Case reports/series on type 1 diabetes mellitus (T1DM) after COVID-19 vaccine, regarding both new onset and disease worsening, are briefly shown in **Table 1** (for complete data, see **Supplementary Table S1**).

Acute onset T1DM following COVID-19 vaccine was reported in 11 case reports/case series (62–72) concerning 15 patients. Median age was 50 years, and eight of 15 were women. T1DM occurred after mRNA-based vaccine (Pfizer/BioNtechBNT162b2 or Moderna mRNA-1273) in 13 of 15 cases, in one case after CoronaVac (65) and one after Vaxzevria ChAdOx1-S (70). Median time to onset of symptoms was 6 days, with a wide range from 3 days (63) to 2 months (71). Most common symptoms were fever, nausea, abdominal pain, fatigue, polydipsia, polyuria, and weight loss. Anti-glutamic acid decarboxylase antibodies and/or insulin autoantibodies were found in nine patients (62, 64, 67, 70, 71). Five patients (64–68) were found to have HLA haplotypes known to confer T1DM risk.

Diabetic ketoacidosis (DKA) in patients with known T1DM was reported in two case series (73, 74) and one case report (75), including five patients (four women and one man; median age was 25 years). In one case, DKA occurred less than 12h after the first dose of Pfizer/BioNTech BNT162b2 (73) in a patient who had consumed approximately 20 g of alcohol on the night before vaccination. The other four cases occurred, with a variable latency between 12h and 6 days, after the second dose of Moderna mRNA-1273 (75), Pfizer/BioNTech BNT162b2 (73), Vaxzevria ChAdOx1 (74) and Covaxin (BBV152-inactivated whole virion) (74). All patients were successfully treated with intravenous fluids and insulin. Furthermore, Infante et al. (76) reported the case of a young T1DM man, previously in optimal glycaemic control with only low basal insulin daily doses, who suffered from a transient deterioration of glucose control in the first days after both Pfizer/BioNTech BNT162b2.

Finally, some studies investigated the short-term effects of COVID-19 vaccination on blood glucose in individuals with T1DM, as shown in **Supplementary Table 2**.

Retrospective studies on patients with T1DM (77, 78) reported that COVID-19 vaccination might be associated with a temporary perturbation of blood glucose levels, especially in patients under oral hypoglycaemic drugs plus insulin and when HbA1c was lower, without differences between vaccine type (mRNA and viral vector).

Aberer and colleagues (79) prospectively investigated the impact of COVID-19 vaccination on time spent in different glycaemic ranges in 58 people with T1DM. No significant differences in time in range were observed, but a worsening of blood glucose was observed concurrently with general side effects after the vaccine.

Three further studies (80–82) retrospectively reported no significant differences in time in range between after and before any vaccination dose.

### Reproductive system and COVID-19 vaccine

#### Female reproductive system

As regards the ovarian function in relation to COVID-19 vaccines, a few studies have been reportedwith different endpoints, as shown in **Supplementary Table 2**.

As far as follicular function is concerned, Mohr-Sasson and colleagues (83) analyzed anti- Müllerian hormone (AMH) levels as an ovarian reserve index in 132 fertile women (mean age was 29 years) before and after the mRNA vaccine. They found that interpersonal difference in plasma AMH levels was not significant between vaccinated and non-vaccinated women. Another study by Bentov et al. (84) assessed whether the immune response to the Pfizer/BioNTech BNT162b2 or virus infection affected ovarian follicles’ function in 32 women undergoing *in vitro* fertilization (IVF) (mean age was 33.7 years), and no effect was found.

Concerning possible ovarian cycle alterations, partially conflicting results were observed.

Indeed, Muhaidat et al. (85) performed a cross-sectional study, *via* online interviews, on 2,269 fertile young women (median age was 34.3 years) undergone to any type of COVID-19 vaccines. Some menstrual abnormalities were self-reported in 66.3% of cases, spontaneously resolving within 2 months. Mean menstrual duration and menstrual cycle length increased of 1 day after vaccine. Symptoms occurred after the first dose in 46.7% of cases and after the second dose in 32.4% of cases, without any differences between the types of vaccine. Of note, almost one-third of the patients had experienced menstrual abnormalities during the COVID-19 pandemic before vaccination.

Similarly, a cohort study performed by Edelman et al. (86) followed six consecutive menstrual cycles (three pre-vaccine and three post-vaccine) in 3,959 women, aged 18–45 years, in order to identify possible vaccine related changes in menses. The vaccinated group included women who had received both mRNA vaccines and viral vector vaccine. The vaccinated cohort experienced 0.64 days increase in the length of their menstrual cycles (compared with pre-vaccinal cycles). Considering patients who received both doses within the same menstrual cycle (358), 38 women (10.6%) experienced a change in cycle length of 8 days or more compared with the unvaccinated control group. These alterations, however, disappeared after two menstrual cycles post-vaccination.

Conversely, Laganà and colleagues (87) performed a cross-sectional study on 164 fertile young women *via* self-administered questionnaire. They found that the main cycle length alteration after COVID-19 vaccine (any type) was 1–5 days shortening of the time between menstruations, both after first and second dose, with a slightly higher prevalence after the second dose (60–70%). Menstrual irregularities were self-resolving within 2 months.

More recently, Lessans et al. (88), reported menstrual cycle alterations in almost 40% of 219 women aged 18-50 years who had undergone mRNA-based vaccine in the 3 months following the vaccine shots.

Finally, Lee et al. (89) retrospectively confirmed these menstrual alterations in a large observational study on 39,129 women (median age was 33 years).

#### Male reproductive system

Male fertility after COVID-19 vaccination has been assessed in one case report (90) (**Supplementary Table S1**) and 11 clinical studies (91, 93–102), as shown in **Supplementary Table 2**.

As for the case report (90), sperm parameters were analyzed after three doses of mRNA vaccine in a 43-year-old man with ankylosing spondylitis under therapy with anti-inflammatory drugs, and no detrimental impact was observed.

Concerning clinical studies, most papers (90, 93–102) evaluated the impact of different type of COVID-19 vaccines on sperm parameters, whereas one article (91) focused on risk of genital infections.

In detail, five studies retrospectively analyzed the potential impact of COVID-19 vaccine (both mRNA, inactivated and viral vector ones) on sperm parameters, three of them in men undergoing fertility treatments (94, 96, 100) and two in healthy semen donors (99, 101). Overall, sperm parameters showed no significant changes after vaccination, except a slight decrease in sperm volume observed by Safrai et al. (96) in patients undergoing IVF treatment, and a lower concentration and motility reported by Gat et al. (99), but the latter alterations spontaneously recovered at a subsequent control.

Moreover, five prospective studies were performed, four of which on healthy young volunteers/sperm donor men (93, 95, 97, 98), and one on men undergoing IVF (for female infertility factors) (102). Overall, no significant alterations of sperm parameters were observed, except in the study by Abd et al. (102), in which they observed a significant reduction of total and progressive sperm motility after the vaccine shot, albeit still in normal range.

Finally, a retrospective large cohort study (91) compared vaccinated and unvaccinated men to assess the risk of developing orchitis and/or epididymitis, and it showed that men who received any type of COVID-19 vaccine had a significantly reduced risk of infections compared with unvaccinated men.

To date, there are no data regarding a negative effect of COVID-19 vaccination on male hypogonadism, testosterone levels and sexual dysfunction.

#### Couple fertility

The impact of COVID-19 vaccination on couple fertility was evaluated in five studies, four of which (13, 103–105) concerning couples undergoing IVF treatments, and one (106) regarding couples trying to conceive naturally (**Supplementary Table 2**). Overall, no differences were observed in fertility rates and in pregnancy outcomes in couple undergoing IVF. As far as spontaneous conceive is concerned (106), no effects were observed after COVID-19 vaccine, compared with SARS-CoV-2 infection, which, on the contrary, was associated with a transient reduction of fecundability in male subjects.

## Discussion

Vaccines are one of the most important advances in medical science and are the most effective method to prevent severe manifestations associated with various infections. However, since they are employed in healthy people, possible adverse effects are of great clinical interest (107). Vaccination campaigns against COVID-19 began more than 1 year ago, and the efficacy of COVID-19 vaccines at 5 months or longer after being fully vaccinated is now about 90% (95% CI, 87–92%) (8, 108, 109). Despite the increasing effectiveness evidence, the occurrence of adverse reactions after vaccines administration may elicit some concern, especially due to the spread misinformation about COVID-19 vaccination, such as infertility in men, which are not substantiated by the data available (3).

In the present review, we summarized the available data concerning COVID-19 vaccine and potential endocrine adverse effects in order to clarify and, possibly, quantify the risk for endocrinopathies after COVID-19 vaccination.

Most findings concern thyroid disorders, especially SAT and Graves’ disease after COVID-19 vaccine, as recently pointed out also by Jafarzadeh and colleagues (9).

With respect to SAT, the findings concerning its occurrence after COVID-19 vaccination are in substantial agreement with the demographic features of the cases previously reported in general population, such as a clear preference for female sex and age between 40 and 50 years (110).

Moreover, as for SAT following other causes (110), SAT occurring after COVID-19 vaccine was generally self-limited, leading to permanent hypothyroidism in about 5% of patients.

Concerning Graves’ disease, there was a clear prevalence of young female patients, in agreement with literature findings (111), being the female to male ratio about 3:1. Fortunately, as for SAT, a favorable outcome was observed in nearly all cases after proper treatment.

Regarding SAT pathogenesis, it is commonly accepted that a recent viral infection may be a triggering agent in SAT development in genetically susceptible individuals (112). Some infections have been associated with SAT occurrence, such as measles, mumps, coxsackie, rubella, adenovirus (112), and COVID-19 infection (113). Also, Graves’ disease onset has been associated with viral infections (111) and several cases of Graves’ disease have been reported after COVID-19 infection (114). Actually, it has been shown that thyroid cells express ACE2, which may facilitate COVID-19 infectivity and lead to a direct attack to the thyroid tissue, with subsequent gland dysfunction (115–117).

More interestingly, SAT has been reported also after vaccinations, such as influenza, human papillomavirus, and hepatitis B vaccines (9). Conversely, the risk of Graves’ disease occurrence after vaccinations has rarely been evaluated (33).

The mechanism by which COVID-19 vaccine may cause SAT and Graves’ disease is still unclear due to limited data.

Possible pathophysiological mechanisms include molecular mimicry, whereby antibodies directed against SARS-CoV-2 proteins cross-react with thyroid antigens and may trigger an autoimmune response in susceptible people (118). An alternative is represented by vaccine-induced higher viscosity status, which may cause an abnormal increase in thyroid hormone levels (119).

Furthermore, both SAT and Graves’ disease have been previously reported as part of the autoimmune/inflammatory syndrome in response to adjuvants (ASIA) (120). ASIA syndrome was firstly described in 2011 by Schoenfeld (121) to include clinical entities which shared similar signs and symptoms together with a previous exposure to adjuvants. Adjuvants exert an important role in vaccines and drugs, since their ability to increase the immunogenicity of the active ingredient is mandatory to achieve an adequate immune response (122). On the other side, adjuvants can induce the formation of autoantibodies. Even if the mRNA-based COVID-19 vaccines, which were more frequently used in patients who developed thyroid issues (both SAT and Graves’ disease), do not contain adjuvants, the mRNA itself could act as an adjuvant, being highly immunostimulatory (123); moreover, it is not clear if the lipid nanoparticles employed to preserve mRNA stability may act as adjuvant (122).

Furthermore, specific HLA haplotypes, such as HLA-B35, may have a role in the susceptibility to SAT in general population (124); interestingly, the association between vaccine-related SAT development and HLA-B35 was found in a recent study (26) even if further data are needed to confirm this relationship.

Taken all the above considerations into account, despite the increasing reports regarding SAT and Graves’ disease, vaccination against COVID-19 is still strongly encouraged, since their occurrence is rare and they are generally easily manageable and/or self-limited.

On the other side, when considering adrenal gland, the possible side effects after COVID-19 vaccine were extremely rare, but more severe than thyroid dysfunction. In fact, of the 16 case reports described so far, nine were about adrenal haemorrhage in the setting of immune thrombotic thrombocytopenia (ITT), and two patients eventually died due to the severity of clinical manifestations. Adrenal haemorrhage developed within 2 weeks and in all cases followed adenoviral vector vaccine administration.

As for the mechanisms by which an adenoviral vector vaccine may cause ITT, it has been hypothesized that there may be a reaction between the cationic PF4 and the anionic free DNA contained in the recombinant vaccine (45, 125). However, the underlying pathways by which COVID-19 vaccination may stimulate anti-PF4 antibody production need further examination.

Concerning the pituitary gland, very few cases of post-vaccination pituitary gland dysfunction have been reported since the beginning of COVID-19 vaccination campaign. So far, data are too sparse and heterogeneous to draw any conclusions about the possible pathogenesis of pituitary dysfunction after COVID-19 vaccination. However, several case reports about COVID-19–induced damage to hypothalamus and pituitary have been observed, such as panhypopituitarism (126), central diabetes insipidus (127) and SIADH resulting in hyponatremia (128, 129); significantly, the expression of ACE2 in the hypothalamus has been identified (92), and the SARS-CoV-2 genome has been detected in the cerebrospinal fluid of a patient with COVID-19, thereby confirming that SARS-CoV-2 can infiltrate into the brain including the hypothalamus and pituitary gland (130). In addition, an autoimmune mechanism triggered by vaccination could play a role in the pathogenetic pituitary damage (as autoimmune hypophysitis). In detail, anti-pituitary antibodies can primarily target corticotropic cells, with consequent isolated secondary hypoadrenalism after vaccination (58).

The possible link between vaccinations and the occurrence of T1DM has been investigated for many different vaccines to date, such as vaccines against influenza virus A/H1N1 (131), Rotavirus (132), human papilloma virus (HPV) (133), with the general agreement that vaccinations are not associated with increased risk of islet autoimmunity or T1DM (134, 135). To date, only few cases of post-COVID-19 vaccination acute diabetes onset have been reported, and in almost all patients a pre-existing genetic predisposition was confirmed (64–68). Therefore, these findings are too few to draw any conclusions about the possible pathogenetic mechanism.

Even when considering already diabetic patients, there are only few reports of DKA after COVID-19 vaccines (regardless of vaccine type), which proved to be easily manageable in all cases. Moreover, the available retrospective and prospective clinical studies showed that COVID-19 vaccines led to none or only temporary perturbations of blood glucose levels (7).

To date, clinical data support the general safety of COVID-19 vaccinations in T1DM patients, with the caveat that patients should be counselled and prepared for possible temporary glycaemic alterations following the vaccine (77). Encouraging vaccination is crucial, since it has been proven that COVID-19 infection may cause islet cells degeneration (7) and that both patients with T1DM and type 2 diabetes mellitus (T2DM), compared with healthy people, carry a worse prognosis in case of Sars-CoV-2 infection, especially in case of poor metabolic control (136).

Concerning female reproductive function related to COVID-19 vaccinations, the main worth mentioning findings are related to the possible and transient slight changes in menstrual cycle length (85–89), even if it is known that several factors may affect cycle length, such as infections, anxiety and hormonal changes, since stressors may activate the hypothalamic-pituitary-gonadal axis, disrupting of the regularity of hormone release (85).

Conflicting results regarding the impact of COVID-19 infection on semen quality have been reported (137). As ACE2 is expressed in spermatogonia and Sertoli and Leydig cells (138), there was concern about the fact that the virus could affect male fertility. Original studies included about 500 semen analyses from men infected with SARS-CoV-2, and some alterations were found in approximatively one third of cases, especially in patients with severe COVID-19 (138). Hence, these findings elicited some concern also about the possible role of COVID-19 vaccination on male fertility (137, 139). However, according to the published data, overall, no significant adverse effects of COVID-19 vaccines on male fertility have been reported so far, and no detrimental effect on both semen quality and fertility outcomes were observed in healthy donors (93, 95, 98, 99, 101), infertile men (94, 96) and couples undergoing assisted reproduction treatments (103, 104) or trying to conceive naturally (106). Of note, COVID-19 vaccination was shown to have a protective role on orchitis and epididymitis (91), which are known risk factor for male infertility (140).

Therefore, the concern regarding the impact of COVID-19 vaccination on male infertility appear as unfounded (141); instead, considering the potential impact of disease on male fertility, vaccination should be recommended (138).

Finally, as different types of COVID-19 vaccines might have different side effects, we tried to figure out a possible relationship between specific endocrine side effects and vaccine types, but we could not obtain clear evidence concerning these hypothetical associations, considering that most of the data came from case reports. Noteworthy, the only likely correlation was about vascular complications following adenoviral vector vaccine administration, but this issue was shown only for adrenal gland, and due to the scant data, we could not generalize this finding nor for adrenal gland or for other endocrine glands in case of adenoviral vector vaccine.

## Conclusions

We summarized the available reports and studies regarding COVID-19 vaccines and potential endocrine adverse effects, showing that their occurrence is generally rare and with benign outcome, being thyroid alterations the most common—but generally easily manageable—findings.

Overall, these data do not call into question the safety and the efficacy of available COVID-19 vaccines, which are relevant and confirmed by multiple studies.

Data on endocrine adverse effects, albeit increasing, are not strong enough to draw any clear conclusion about their causal relationship with vaccine administration, since they are based mainly on case-reports; therefore, further clinical and preclinical studies are needed to clarify the possible mechanisms underlying these manifestations.

## Data availability statement

The original contributions presented in the study are included in the article/**Supplementary Material**. Further inquiries can be directed to the corresponding author.

## Author contributions

CC conceived the project. LP and CC provided supervision and project administration. EG, FB, PF, MG, MC, CB and ED did the literature search. LP, EG, FB, PF, MG, MC, CB and ED wrote the original draft. EG, FB and PF cured the supplementary content. MR, AF and LP reviewed and edited the manuscript. All authors contributed to the article and approved the submitted version.

## Conflict of interest

The authors declare that the research was conducted in the absence of any commercial or financial relationships that could be construed as a potential conflict of interest.

## Publisher’s note

All claims expressed in this article are solely those of the authors and do not necessarily represent those of their affiliated organizations, or those of the publisher, the editors and the reviewers. Any product that may be evaluated in this article, or claim that may be made by its manufacturer, is not guaranteed or endorsed by the publisher.
