# Supplementary material for: Endocrine system after 2 years of COVID-19 vaccines: A narrative review of the literature
Source: Front Endocrinol (Lausanne). 2022 Nov 10;13:1027047. doi: 10.3389/fendo.2022.1027047 (PMC9685624; doi:10.3389/fendo.2022.1027047)
Supplement: Supplementary file 1 [file Table_1.pdf]

Supplementary Table 1. Case reports concerning endocrine adverse effects after COVID-19 vaccine.

Thyroid Gland

Subacute Thyroiditis

Author, year	Study design	Patient(s)	Type of vaccine	Latency between vaccine and symptoms	Clinical presentation	Treatment	Outcome/Conclusions
Iremli et al, 2021(1)	Case series	W, 35 years, no medical history	Sinovac Biotech Coronavac	4 days after 2nd dose	Anterior cervical pain, fever, palpitations and fatigue	Methylprednisolone and propranolol	Neck pain and palpitation complaints disappeared within a day, TFTs were normalized within 4 weeks
		W, 34 years, mild COVID infection 5 months prior	Sinovac Biotech Coronavac	4 days after 1st dose	Anterior cervical pain, fever, fatigue and palpitations	Methylprednisolone 16 mg/day and propranolol 25 mg/12h	Myalgia and neck pain reappeared during methylprednisolone tapering; complaints disappeared after 8 weeks
		W, 37 years, no medical history	Sinovac Biotech Coronavac	7 days after 1st dose	Anterior cervical pain	No treatment	Neck pain disappeared and TFTs were normalized within 8 weeks
Franquemont et al, 2021(2)	Case report	W, 42 years, no medical history	Pfizer/BioNtech BNT 16b2	5 days after 1 st dose	Anterior cervical pain and palpitations	Prednisone 40 mg/day and propranolol 20 mg as needed for symptoms	Rapid improvements of symptoms, after 1 month; TFTs suggested subclinical hyperthyroidism
Pujol et al, 2021(3)	Case series	W, 38 years, Asthma and Gilbert disease	Moderna mRNA-1273	8 days after 1st dose	Anterior cervical pain, palpitations, distal tremor, axillar and inguinal bilateral ganglionic reaction	Prednisone, propranolol and Ibuprofen	Improvements of symptoms within a week, TFTs were normalized
		W, 32 years, type 1 diabetes	Pfizer/BioNtech BNT 16b2	10 days after 1st dose	Palpitations and insomnia	No treatment	Overt hypothyroidism 8 weeks later, treatment with levothyroxine was started.
Saygılı et al, 2021(4)	Case report	W, 38 years, no medical history	Sinovac Biotech Coronavac	14 days after 2nd dose	Neck swelling, pain, fatigue, loss of appetite and sweating	Naproxen sodium 275 mg/12h and propranolol 20 mg/12 h	Improvements of symptoms, overt hypothyroidism 1 month later, treatment with levothyroxine was started
Sahin Tekin et al, 2021(5)	Case report	M, 67 years, hypertension and atrial extrabeats	Sinovac Biotech Coronavac	18 days after 2nd dose	Fever, weight loss and neck and ear pain	Ibuprofen 800 mg/day	Neck pain intermittently continued for a while, TFTs were normalized within 2 months
Bornemann et al, 2021(6)	Case series	W, 26 years, no medical history	Vaxzevria ChAdOx1	14 days after 1st dose	Cervical pain radiated to both ears	Ibuprofen 600 mg/day, then prednisolone 50 mg/day	Improvements of symptoms within 2 weeks, TFTs were normalized within 6 weeks
		W, 49 years, no medical history	Moderna mRNA-1273	14 days after 1st dose	Headaches and difficulty in concentrating, then a right cervical sore throat	Ibuprofen 600 mg/day, then diclofenac 50 mg/day due to gastrointestinal intolerance	Improvements of symptoms within 2 weeks, the patient discontinued the treatment and displayed thyrotoxicosis with positive anti-TPO after 4 weeks; symptoms improved after treatment with prednisolone 20 mg/day
Plaza-Enriquez et al, 2021(7)	Case report	W, 42 years, right hemicolectomy for adenocarcinoma of colon followed by adjuvant chemotherapy, paroxysmal supraventricular tachycardia, iron deficiency anemia, chemotherapy-induced neuropathy and lumbar radiculopathy	Moderna mRNA-1273	5-6 days after 2nd dose	Bilateral earache radiating down to the lateral and anterior neck and bilateral lower jaw, fever, fatigue, malaise, anorexia, myalgia, dysphagia, dysphonia, dyspnea cough, rhinorrhea, lacrimation, and ear discharge	NSAIDs	Cervical pain and TFTs were improved within 2 months

Siolos et al, 2021(8)	Case report	W, 51 years, no medical history	Pfizer/BioNtech BNT 16b2	14 days after 1st dose	Nausea, mild anterior neck pain, and fever up to 38.2°C	Methylprednisolone 16 mg/day	Symptoms and TFTs were improved within 2 months
	Case report	W, 39 years, no medical history	Vaxzevria ChAdOx1	21 days after unknown dose	NA	No treatment	TFTs were improved within 2 months
Schimmel et al, 2021(9)	Case report	W, 57 years, no medical history	Pfizer/BioNtech BNT 16b2	1 day after 2nd dose	Cervical pain and swelling	Ibuprofen and propranolol	NA
Oyibo, 2021(10)	Case report	W, 55 years, well controlled asthma	Vaxzevria ChAdOx1	21 days after 1st dose	Cervical pain and swelling, sore throat, headache, palpitations	NSAIDs, paracetamol and propranolol	Overt hypothyroidism 6 weeks later, treatment with levothyroxine 50 mcg/daily was started
Ratnayake et al, 2021(11)	Case report	M, 75 years, no medical history	Vaxzevria ChAdOx1	14 days after 1st dose	Cervical pain and fever	Ibuprofen	Symptoms gradually resolved, TFTs were normalized within 1 month
Chatzi et al, 2021(12)	Case report	W, 35 years, no medical history	Pfizer/BioNtech BNT 16b2	12 days after 1st dose	Cervical pain radiated to jaw and ear, fatigue, palpitations	Prednisolone	NA
	Case report	W, 32 years, no medical history	Pfizer/BioNtech BNT 16b2	4 days after 2nd dose	Cervical pain, fatigue	Prednisolone	NA
Jeeyavudeen et al, 2021(13)	Case report	W, mid age, no medical history	Pfizer/BioNtech BNT 16b2	14 days after 2nd dose	Thyroid painful swelling, poor sleep, night sweats, weight loss, hyperdefaecation	NSAIDs	Symptoms disappeared within 6 weeks, TFTs were normalized within 8 weeks
Kyriacou et al, 2021(14)	Case report	W, 40 years, NA medical history	Pfizer/BioNtech BNT 16b2	12 hours after 2nd dose	Anterior cervical pain, malaise, bony aches, emotional lability, hyperhidrosis, palpitations	Prednisolone 40mg/day, propranolol	Symptoms disappeared after 24-48 hours, TFTs were normalized within a month
Soltanpoor et al, 2021(15)	Case report	W, 34 years, no medical history	Bharat Biotech BBV512	5-7 days after 1st dose	Mild fever, palpitation, anterior cervical pain	Prednisolone 15 mg/day and propranolol 20 mg/12 h	Symptoms disappeared within 2 weeks, TFTs were normalized within 7 weeks
Lee et al, 2021(16)	Case report	W, 39 years, NA medical history	Vaxzevria ChAdOx1	4 days after 2nd dose	Cervical pain	NA	NA
	Case report	W, 73 years, NA medical history	Vaxzevria ChAdOx1	11 days after 1st dose	Cervical pain and fever	NA	NA
Khan et al, 2021(17)	Case report	W, 42 years, asymptomatic COVID-19 infection 3 months prior	Pfizer/BioNtech BNT 16b2	4 days after 2nd dose	Palpitations, fever and left cervical pain	Prednisolone 30 mg/day, propranolol 80 mg/day and ibuprofen 400 mg/8 h	Symptoms improved in few days, TFTs were normalized within 4 weeks
Leber et al, 2021(18)	Case report	W, 32 years, NA medical history	Sinovac Biotech Coronavac	12 hours after 2nd dose	Low visual acuity and pain on movement of the left eye and headache	Methylprednisolone 1 g intravenously for 6 days	Symptoms improved and TFTs normalized 3 days after discharge
Sözen et al, 2021(19)	Case series	M, 41years, no medical history	Pfizer/BioNtech BNT 16b2	8 days after 1st dose	Cervical pain, palpitation, fatigue	Acetylsalicylic acid 500 mg/6 h and propranolol 20 mg/12 h	Symptoms improved within few weeks, overt hypothyroidism, which improved over the next several months
		W, 40 years, no medical history	Pfizer/BioNtech BNT 16b2	6 days after 2nd dose	Cervical pain, palpitation, sweating	Acetylsalicylic acid 500 mg/6 h and propranolol 20 mg/12 h	Symptoms improved within 2 weeks, TFTs were normalized within 1 month
		M, 40 years, smoker for 15 years	Pfizer/BioNtech BNT 16b2	4 days after 1st dose	Cervical pain, fatigue, nervousness	Ibuprofen 600 mg/8 h	NA
		W, 26 years, no medical history	Pfizer/BioNtech BNT 16b2	6 days after 1st dose	Cervical pain	Acetylsalicylic acid 500 mg/6 h and propranolol 20 mg/12 h	Symptoms improved within 2 weeks, called for outpatient follow-up 1 month later in terms of the risk of developing hypothyroidism
		W, 44 years, Hashimoto's thyroiditis and hypothyroidism well controlled with levothyroxine 50 mcg/daily	Pfizer/BioNtech BNT 16b2	6 days after 1st dose	Cervical pain, headache, palpitation, sweating and tremor	Ibuprofen 600 mg/8 h	Symptoms improved within the next weeks, TFTs were normalized within the next weeks

Pandya et al, 2021(20)	Case series	M, 37 years, prediabetes and dyslipidemia	Moderna mRNA-1273	15 days after 1st dose	Fever, cervical pain, tachycardia, palpitations, proptosis	Propranolol, ibuprofen and then prednisone	Symptoms improved after prednisone was started
		M, 35 years, no medical history	Pfizer/BioNtech BNT 16b2	10 days after 1st dose	Cervical pain, palpitations	Propranolol and ibuprofen	Symptoms disappeared in few days, TFTs improved within 2 weeks
		W, 41 years, no medical history	Pfizer/BioNtech BNT 16b2	20 days after 2nd dose	Tachycardia, palpitations	Diltiazem and ibuprofen	NA
Sigstad et al, 2021(21)	Case report	W, in her thirties, concurrent diagnosis of papillary thyroid carcinoma	Pfizer/BioNtech BNT 16b2	6 days after 1st dose	Feeling a lump on throat	No treatment for SAT	Hemithyroidectomy followed by complementary hemithyroidectomy, and then radioactive iodine therapy
González López et al, 2021(22)	Case series	W, 32 years, no medical history	Pfizer/BioNtech BNT 16b2	3 days after 2nd dose	Cervical pain, fever, fatigue	Naproxen	TFTs were normalized within 4 weeks
		W, 33 years, no medical history	Vaxzevria ChAdOx1	1 day after 1st dose	Cervical pain radiated to ear, fever, fatigue, tachycardia, weight loss	Ibuprofen and propranolol	Asymptomatic overt hypothyroidism after 2 months
Rebollar, 2021(23)	Case report	W, 53 years, no medical history	Convidecia AD5-nCOV	15 days after 1st dose	Malaise, asthenia, fever, arthralgia and myalgia	NSAIDs	Symptoms improved, overt hypothyroidism after 4 weeks
Ippolito et al, 2022(24)	Case series	W, 41 years, no medical history	Pfizer/BioNtech BNT 16b2	15 days after 1st dose	Neck pain radiating to ear and jaw, fast heartbeat, sweating	Propranolol 40 mg/day and prednisone 25 mg/day tapered and discontinued after 8 weeks	Transient hypothyroidism not treated and spontaneously resolved
		W, 31 years, seronegative hypothyroidism, well controlled with levothyroxine 50 mcg daily.	Pfizer/BioNtech BNT 16b2 (1st dose Vaxzevria ChAdOx1)	28 days after 2nd dose	Severe neck pain radiating to ear, fast heartbeat, sweating, headache	LT4 discontinuation, propranolol 40 mg/day, ibuprofen, prednisone 25 mg/day tapered and discontinued after 10 weeks	Mild symptomatic and biochemical relapse after steroid discontinuation
		W, 64 years, no medical history	Pfizer/BioNtech BNT 16b2	18 days after 2nd dose	Moderate cervical pain, mild fever, fatigue, palpitations, sweating	Prednisone 25 mg/day tapered and discontinued after 4 weeks	Improvements of symptoms, TFTs were normalized within 4 weeks
Yorulmaz et al, 2022(25)	Case series	W, 47 years, no medical history	Sinovac Biotech Coronavac	21 days after 1st dose	Cervical pain, headache, tremors, sweating	NSAIDs	Improvements of symptoms, TFTs were normalized within 1 month
		M, 67 years, no medical history	Sinovac Biotech Coronavac	19 days after 2nd dose	Cervical pain, weight loss, fever, tachycardia	NSAIDs and betablockers	Improvements of symptoms, TFTs were normalized within 2 months
		W, 62 years, no medical history	Pfizer/BioNtech BNT 16b2	30 days after 2nd dose	Cervical pain	NSAIDs	Improvements of symptoms, TFTs were normalized within 2 months
		M, 44 years, no medical history	Pfizer/BioNtech BNT 16b2	15 days after 1st dose	Cervical pain, weight loss, fever, sweating	NSAIDs	Overt hypothyroidism, treatment with levothyroxine was started
		M, 26 years, no medical history	Pfizer/BioNtech BNT 16b2	37 days after 2nd dose	Cervical pain, weight loss, fever, tremors, myalgia	NSAIDs	Improvements of symptoms, TFTs were normalized within 1.5 months
		W, 37 years, previous Grave's disease	Sinovac Biotech Coronavac	15 days after 2nd dose	Cervical pain, dysphagia	NSAIDs	Overt hypothyroidism, treatment with levothyroxine was started
		W, 39 years, no medical history	Pfizer/BioNtech BNT 16b2	18 days after 1st dose	Weight loss, tachycardia	NSAIDs and betablockers	Improvements of symptoms, TFTs were normalized within 2.5 months
		W, 40 years, no medical history	Pfizer/BioNtech BNT 16b2	15 days after 2nd dose	Cervical pain, fever	NSAIDs	Improvements of symptoms, TFTs were normalized within 2 months
		M, 29 years, no medical history	Pfizer/BioNtech BNT 16b2 (1st and 2nd dose with Sinovac Biotech Coronavac)	15 days after 3rd dose	Cervical pain	NSAIDs	Improvements of symptoms, TFTs were normalized within 2 months

		W, 73 years, previous SAT 20 years before	Sinovac Biotech Coronavac	30 days after 2nd dose	Cervical pain, tachycardia	NSAIDs and betablockers	Overt hypothyroidism, treatment with levothyroxine was started
		W, 30 years, no medical history	Pfizer/BioNtech BNT 16b2	30 days after 2nd dose	Cervical pain	NSAIDs	Overt hypothyroidism, treatment with levothyroxine was started
Patel et al, 2022(26)	Case report	M, 48 years, NA medical history	NA	7 days after 2nd dose	Right cervical swelling, fever, palpitations and weight loss	NSAIDs and prednisone 40 mg/day	Symptoms disappeared after 24 hours
Pla Peris et al, 2022(27)	Case series	M, 57 years, NA medical history	Moderna mRNA-1273	10-14 days after 1st dose	No neck pain, no swelling, mild fever,<br>asthenia, weight loss, palpitation	NSAIDs	Subclinical hypothyroidism
		M, 67 years, NA medical history	Moderna mRNA-1273	10-14 days after 1st dose	Neck pain radiating<br>to ears, asthenia, mild fever, tachycardia	NSAIDs	TFTs were normalized
		M, 47 years, NA medical history	Pfizer/BioNtech BNT 16b2	10-14 days after 1st dose	Neck pain radiating<br>to ears, asthenia, mild fever, tachycardia	NSAIDs	Subclinical hypothyroidism
Bostan et al, 2022(28)	Case series	M, 61 years, diabetes mellitus, hypertension priori COVID-19 infection	Sinovac Biotech Coronavac	6 days after 1st dose	Cervical swelling in the anterior area and pain radiating to the jaw, palpitations, fever	Ibuprofen 200 mg/day and propranolol 20 mg/12 h	Symptoms disappeared, asymptomatic subclinical hypothyroidism within a month
		W, 32 years, breastfeeding for 10 months	Pfizer/BioNtech BNT 16b2	3 days after 1st dose	Cervical pain, fever, arthralgia	Methylprednisolone 16 mg/day and propranolol 20 mg/12 h	Symptoms improved and TFTs were normalized within 3 weeks
Jhon et al, 2022(29)	Case report	M, 34 years, NA medical history	Moderna mRNA-1273	5 days after 1st dose	Feeling a lump when swallowing, cervical pain, weight loss, fatigue, headache, muscle weakness, tachycardia	Prednisone 30 mg/day	Symptoms rapidly improved, TFTs were normalized after 55 days
Vasileiou et al, 2022(30)	Case report	W, 36 years, ulcerative gastritis, intraocular hypertension, endometriosis	Pfizer/BioNtech BNT 16b2	10 days after 1st dose	Fatigue, palpitations, and cervical pain that radiated to the ear and jaw	No treatment	The 2nd dose of the vaccine was administrated 10 days later, the symptoms recurred. Treatment was done with paracetamol and ibuprofen but the symptoms were worsened and TFTs were abnormal. Treatment with methylprednisolone improved symptoms and 2 weeks later TFTs were normalized
Das et al, 2022(31)	Case report	W, 47 years, NA medical history	Vaxzevria ChAdOx1	14 days after 1st dose	Fever, cervical pain radiated to the jaw, weight loss, difficulty in swallowing, restlessness, tachycardia	Propranolol 40 mg/day	Symptoms disappeared and TFTs were normalized within 8 weeks
Bahçecioğlu et al, 2022(32)	Case series	W, 82 years, NA medical history	Sinovac Biotech Coronavac	14 days after 2nd dose	Fever, tremor, weight loss	NSAIDs and β-blockers	NA
		W, 79 years, NA medical history	Sinovac Biotech Coronavac	7 days after 2nd dose	Fever, tremor, anorexia, asthenia, weight loss	NSAIDs and β-blockers	NA
		W, 41 years, NA medical history	Pfizer/BioNtech BNT 16b2	28 days after 2nd dose	Cervical pain	NSAIDs	NA
		W, 28 years, NA medical history	Sinovac Biotech Coronavac	77 days after 2nd dose	Cervical pain, weight loss	NSAIDs	NA
		W, 41 years, NA medical history	Pfizer/BioNtech BNT 16b2	28 days after 2nd dose	Cervical pain, palpitations, sweating	Methylprednisolone 24 mg/day and β-blockers	NA
		M, 71 years, NA medical history	Sinovac Biotech Coronavac	84 days after 2nd dose	Cervical pain, asthenia, weight loss	Methylprednisolone 16 mg/day	NA
Pipitone et al, 2022(33)	Case report	W, 49 years, nomedical history	Pfizer/BioNtechBNT 16b2	7 days after 1st dose	Anterior cervical pain, anxiety, dysphagia, fatigue, increased bowel motility, insomnia, night sweat, palpitation, tachycardia, and weight loss	Prednisone, NSAIDs, andβ-blockers	TFTs were normalized 3 weeks after discharge

Stasiak et al, 2022(34)	Case series	M, 51 years, NA medical history	Pfizer/BioNtech BNT 16b2	21 days after 2nd dose	Tachycardia, fever, right anterior cervical pain, excessive sweating	Prednisone	Symptoms rapidly improved
		M, 45 years, previous SAT 20 years before	Pfizer/BioNtech BNT 16b2	21 days after 2nd dose	Symptoms recurred with slightly less severe cervical pain than during the first SAT episode	Prednisone	Complete resolution of US lesions within 3 months
Bennet et al, 2022(35)	Case report	W, 52 years, NA medical history	Vaxzevria ChAdOx1	7 days after 1st dose	NA	Propranolol	Symptoms disappeared and TFTs were normalized within 7 weeks
Kishimoto et al, 2022(36)	Case series	W, 54 years, no medical history	Pfizer/BioNtech BNT 16b2	1 day after 2nd dose	Fever, anterior cervical pain, general fatigue, shortness of breath	Prednisolone 20 mg/day	Symptoms rapidly improved, TFTs were normalized within 4 weeks
		M, 46 years, hypercholesterolemia	Moderna mRNA-1273	10 days after 1st dose	Persistent fever, odynophagia, and bilateral anterior neck pain	Prednisolone 20 mg/day	Symptoms improved, TFTs were normalized within 3 months
Huo et al, 2022(37)	Case report	W, 65 years, diabetes	NA	NA	Swallowing pain of right neck, headache, fever, fatigue	Injecting the thyroid capsule with a mixture of 2% lidocaine (1 ml) and 0.5% dexamethasone (1 ml) for 3 times	Symptoms rapidly improved
Adelmeyer et al, 2022(38)	Case series	W, 36 years, no medical history, smoker	Pfizer/BioNtech BNT 16b2	1 day after 2nd dose	Cervical pain, restlessness, fatigue	Ibuprofen and then prednisolone 30 mg/day	Treatment with prednisolone improved symptoms, TFTs were normalized
		M, 65 years, sigmoid diverticulitis	Vaxzevria ChAdOx1	3 days after 1st dose	Anterior cervical pain, hoarseness	No treatment	Symptoms disappeared, TFTs were normalized within 1 month
Borges Canha et al, 2022(39)	Case report	W, 32 years, no medical history	Vaxzevria ChAdOx1	4 days after 1st dose	Fever, painful swallowing and cervical pain	Prednisolone 40 mg/day	Symptoms improved within few days, TFTs were normalized within 5 months
Oğuz et al, 2022(40)	Clinical study	W, 42 years, nodular thyroid disease	Pfizer/BioNtech BNT 16b2	4 days after 1st dose	NA	NSAIDs	Symptoms disappeared within 14 weeks
		W, 48 years, prior subacute thyroiditis 1 month before	Sinovac Biotech Coronavac	1 day after 2nd dose	NA	Prednisolone 5 mg/day and then 10 mg/day	Symptoms disappeared within 5 weeks
		W, 47 years, systemic lupus erythematosus	Pfizer/BioNtech BNT 16b2	10 days after 1st dose	NA	Occasional paracetamol	Symptoms disappeared within 13 weeks
		W, 72 years, prior subacute thyroiditis	Pfizer/BioNtech BNT 16b2 (1st and 2nd dose with Sinovac Biotech Coronavac, 3rd dose with Pfizer/BioNtech BNT 16b2)	15 days after 4th dose	NA	No treatment	Symptoms disappeared within 5 weeks
		W, 50 years, MNG, prior subacute thyroiditis	Sinovac Biotech Coronavac	1 day after 1st dose	NA	NSAIDs	Symptoms disappeared within 6 weeks
		W, 61 years, MNG	Sinovac Biotech Coronavac	15 days after 2nd dose	NA	Methylprednisolone 16 mg/day	Symptoms disappeared within 20 weeks
		W, 36 years, no medical history	Sinovac Biotech Coronavac	4 days after 2nd dose	NA	Methylprednisolone 16 mg/day	Not in remission
		W, 38 years, no medical history	Sinovac Biotech Coronavac	7 days after 2nd dose	NA	No treatment	Symptoms disappeared within 11 weeks
		W, 38 years, no medical history	Pfizer/BioNtech BNT 16b2	10 days after 1st dose	NA	NSAIDs	Symptoms disappeared within 4 weeks
		W, 38 years, no medical history	Sinovac Biotech Coronavac	13 days after 1st dose	NA	Occasional paracetamol, NSAIDs during relapse	Symptoms aggravated after 2nd dose and disappeared within 12 weeks

		W, 43 years, no medical history	Pfizer/BioNtech BNT 16b2	7 days after 2nd dose	NA	Methylprednisolone 16 mg/day and NSAIDs	Symptoms disappeared within 11 weeks
		W, 60 years, MNG, prior subtotal hemitiroidectomy,undifferentiated connective tissue disease	Pfizer/BioNtech BNT 16b2 (1st and 2nd dose with Sinovac Biotech Coronavac)	3 days after 3rd dose	NA	No treatment	Not in remission
		W, 46 years, no medical history	Pfizer/BioNtech BNT 16b2	0 days after 1st dose	NA	NSAIDs, Methylprednisolone 16 mg/day during relapse	Symptoms aggravated after 2nd dose and disappeared within 18 weeks
		W, 34 years, no medical history	Sinovac Biotech Coronavac	4 days after 1st dose	NA	Methylprednisolone 16 mg/day and methimazole 5 mg/day	Not in remission
		M, 71 years, no medical history	Pfizer/BioNtech BNT 16b2 (1st and 2nd dose with Sinovac Biotech Coronavac)	10 days after 3rd dose	NA	Prednisolone 20 mg/day	Not in remission
Topaloğlu et al, 2022(41)	Case report	M, 41 years, NA medical history	Sinovac Biotech Coronavac	NA days after 3rd dose	Sore throat, cervical pain	Methylprednisolone	Symptoms disappeared; two weeks after the second dose the patients suffered from neck pain and hoarseness, treated with ibuprofen. Overt hypothyroidism, treatment with levothyroxine was started
Bres et al, 2022(42)	Case series	W, 56 years, NA medical history	Moderna mRNA-1273	7 days after 2nd dose	Fatigue, severe weight loss, evening fever (39.5 ◦C) with intense perspiration, diarrhea episodes and tachycardia; anterior neck pain; TFTs consistent with hyperthyroidism; thyroid ultrasound suggestive for subacute thyroiditis	Prednisone	Rapid clinical improvement and thyroid function normalization
		W, 44 years, NA medical history	Pfizer/BioNtech BNT 16b2	5 weeks after 1st dose	Severe anterior right neck pain associated with headache; TSH within normal range, elevated c reactive protein; thyroid ultrasound suggestive for subacute thyroiditis	Prednisone	Her complaints regressed 3 days after treatment initiation, but pain reappeared at the end of the corticosteroid therapy. Reintroduction of prednisone was effective. She received a second dose 4 months after the first without complications
Vu et al, 2022(43)	Case report	W, 38 years, no significant medical history	Vaxzevria ChAdOx1	5 days after 2nd dose	Headache, vertigo, fatigue, palpitations, shortness of breath, small amplitude tremors, and sweating episodes. TFTs showed suppressed TSH, high fT3 and fT4 values, negative anti-thyroid antibodies	Metoprolol	After 3 months of following, all clinical symptoms disappeared.Thyroid hormone tests came back normal
Murashita et al, 2022(44)	Case report	M, 26 years, no significant medical history	Moderna mRNA-1273	Immediately after 2nd dose	Fever, headache, mild neck pain, and palpation. Shortly after, he developed limb weakness and difficulty in walking. TFTs showed suppressed TSH, high fT3 and fT4 values, very low potassium	Prednisolone, potassium chloride	Diagnosed with subacute thyroiditis and thyrotoxic periodic paralysis.<br>On day 160, the TSH level was normalized.
Pi et al, 2022(45)	Case report	W, 32 years, NA medical history	Sinopharm Beijing	3 days after 2nd dose	Anterior neck pain and swelling, palpitations; TFTs showed low TSH, high fT3 and fT4, positive TPOAb and TGAb, negative TRAb and elevated c reactive protein; ultrasound and scintigraphy	Celecoxib and propranolol	Neck pain was relieved 20 days later; hypoechoic areas disappeared 6 months later.<br>The thyroid function turned out to hypothyroidism requiring levothyroxine

							consistent with SAT

Graves' Disease

Author, year	Study design	Patient(s)	Type of vaccine	Latency between vaccine and symptoms	Clinical presentation	Treatment	Outcome/Conclusions
Lee et al, 2021(16)	Case series	W, 46 years, no medical history	Vaxzevria ChAdOx1	1 day after 1st dose	Chest pain and dyspnea	NA	Diagnosed with Graves' disease with heart failure
		W, 73 years, normal thyroid function before	Vaxzevria ChAdOx1	14 days after 2nd dose	Weight loss, dyspnea	NA	Diagnosed with Graves' disease
		M, 34 years	Janssen Ad26.COV2.S	14 days after 1st dose	Weight loss, palpitation	NA	Diagnosed with recurrent Graves' disease
		M, 39 years	Janssen Ad26.COV2.S	14 days after 1st dose	Fever, neck pain	NA	Diagnosed with concurrent Graves' disease and subacute thyroiditis
		M, 33 years	Janssen Ad26.COV2.S	14 days after 1st dose	Both leg weakness	NA	Diagnosed with painless thyroiditis with thyrotoxic periodic paralysis
Pla Peris et al, 2021(27)	Case series	W, 71 years	Pfizer/BioNtech BNT 16b2	60 days after 2nd dose	Weight loss, asthenia, atrial fibrillation	Methimazole	Diagnosed with Graves' disease. Improvement in symptoms, TSHR-Ab remained positive 2 months later;
		W, 42 years	Pfizer/BioNtech BNT 16b2	10-14 days after 1st dose	Weight loss, asthenia, palpitations	Methimazole	Diagnosed with Graves' disease. Improvement in symptoms, TSHR-Ab remained positive 2 months later
		W, 54 years	Moderna mRNA-1273	10-14 days after 2nd dose	Weight loss, asthenia, palpitations	Methimazole	Diagnosed with Graves' disease. Improvement in symptoms
		W,46 years	Pfizer/BioNtech BNT 16b2	50 days after 1st dose	Weight loss, palpitations, irritability	Methimazole	Diagnosed with Graves' disease. Improvement in symptoms
		W, 69 years	Pfizer/BioNtechBNT 16b2	10-14 days after 1st dose	Neck pain, mild fever. Weightloss, palpitation, hand tremor	Methimazole and non-steroidal antinflammatory drugs	Diagnosed with concurrent Graves' disease andsubacute thyroiditis. Improvement in symptoms
Zettinig et al, 2021(46)	Case series	W, 61 years	Pfizer/BioNtech BNT 16b2	35 days after 2nd dose	Sweating, palpitations	Methimazole	Diagnosed with Graves' disease. TFTs were quickly corrected
		W, 38 years	Pfizer/BioNtech BNT 16b2	12 days after 1st dose	Insomnia, nervousness, high sweating	Methimazole	Diagnosed with Graves' disease. TFTs were quickly corrected
Raven et al, 2021(47)	Case report	W, 35 years no medical history	Vaxzevria ChAdOx1	5 days after 1st dose	Palpitations, heat intolerance, hyperphagia, tremor	Carbimazole	Diagnosed with Graves' disease. Still in follow up
Vera-Lastra et al, 2021(48)	Case series	W, 40 years, arterial hyper-tension and a history of COVID-19 8 months	Pfizer/BioNtechBNT 16b2	2 days after 1st dose	Nausea, vomiting, fatigue, insomnia, and palpitations, sinus tachycardia and episodes of paroxysmal atrial fibrillation.	Propranolol, ivabradine, diltiazem, and thiamazole	Diagnosed with Graves' disease. Good response to treatment
		W, 28 years, no medical history	Pfizer/BioNtech BNT 16b2	3 days after 1st dose	Anxiety, insomnia, palpitations and distal tremor	Propranolol and thiamazole	Diagnosed with Graves' disease. Good response to treatment
Lui et al, 2021(49)	Case report	W, 40 years, no medical history	Pfizer/BioNtech BNT 16b2	5 days after 2nd dose	Palpitations and tachycardia	Carbimazole and propranolol	Diagnosed with Graves' disease. Improvement in the thyroid function
Weintraub etal, 2021(50)	Case series	W, 38 years	Moderna mRNA-1273	7 days after 1st dose	Abdominal pain, fever,tachycardia	Methimazole andpropranolol	Diagnosed with Graves' disease. Reported improved energy level and denied recurrence of palpitations or abdominal pain.
		W, 68 years	Pfizer/BioNtech BNT 16b2	28 days after 2nd dose	Pruritic rash	No medication	Diagnosed with Graves' disease. After 6 months, TSH was normalised
		M, 30 years	VaxzevriaChAdOx1	2 days after 2nddose	Palpitations, tremor, weight loss, irritability	Methimazole and atenolol	Diagnosed with Graves' disease. His irritability and restless sleep improved; however, he still endorsed occasional palpitations.

Sriphrapradang . et al, 2021(51)	Case report	M, 70 years, no medical history	Vaxzevria ChAdOx1	2 days after 2nd dose	Weight loss, dyspnea, myalgia, and palpitation	Methimazole	Diagnosed with Graves' disease. Good response to treatment
Pierman et al, 2021(52)	Case report	W, 34 years, no medical history	Pfizer/BioNtech BNT 16b2	10 days after 1st dose	Eyelid swelling, weight loss, tremor, sweating, dyspnea	Thiamazole	Diagnosed with Graves' disease. Still in follow up
Patrizio et al, 2021(53)	Case report	M, 52 years, history of vitiligo vulgaris and 8 years type 2 diabetes mellitus	Pfizer/BioNtechBNT 16b2	28 days after 2nd dose	Weight loss, myalgia, dyspneaand palpitation	Methimazole and atenolol	Diagnosed with Graves' disease. Progressive improvement in symptoms, disappearance of fever and normalisation of thyroid hormones.
Goblirsch et al, 2021(54)	Case report	W, 71 years, history of stage 4 breast cancer in remission, struma ovarii at age 35 years and clinically stable multinodular goitre	Pfizer/BioNtechBNT 16b2	14 days after 2nd dose	Tachycardia, breath shortness, sweating, dizziness, hand tremors, nausea	Methimazole and atenolol	Diagnosed with Graves' disease. Improvement in symptoms. Now clinically stable. Resolved tachycardia and her FT4 and total FT3 levels have returned to baseline.
Sriphrapradang et al, 2021(55)	Case report	W, 30 years, active GD for 3 years, euthyroid with MMI 2,5 mg/day	VaxzevriaChAdOx1	4 days after 1st dose	Palpitations, lost weight of 2 kg despite an increased appetite,	Methimazole (increasing dose from 2.5 to5 mg) and add propranolol	Diagnosed with relapsed Graves' Disease. Thyrotoxic symptoms were improved. TFTs at 1 month after an adjusted methimazole dose remained T3-toxicosis
Rubinstein etal, 2021(56)	Case report	W, 50 years, GD treated with radioactive iodine, hypertension, anxiety	Pfizer/BioNtechBNT 16b2	3 days after 2nddose	Eye irritation, tearing, visual changes, orbital pain, and proptosis of theeyes bilaterally, left more than right.	Intravenous teprotumumab.	Diagnosed with rapidly developing Graves' ophthalmopathy. After the second dose, she had significant improvement in congestive symptoms, in addition to 1mm reduction in proptosis of the OD and 3mm of the left.
Di Filippo etal, 2021(57)	Case series	M, 32 years, no medical history	VaxzevriaChAdOx1	10 days after 2nd dose	Anxiety, tachycardia and palpitations	Methimazole (switched toPTU after rush) and propranolol	Diagnosed with Graves' disease. TFTs normalised after3 months
		M, 35, no medical history	VaxzevriaChAdOx1	5 days after 1st dose	Headache, nausea, asthenia, palpitations, tachycardia, mildeyes-redness and superior palpebral retraction.	Thiamazole and propranolol	Diagnosed with Graves' disease. After three months of therapy his thyroid function and TRAbs levels are currently normal on 5 mg/daily dose of thiamazole. Patient was counselled elsewhere not to undergo the second dose of the vaccine.
Lui et al, 2021(49)	Case report	W, 40 years, history of subclinical hypothyroidism in therapy with thyroxine	Pfizer/BioNtechBNT 16b2	39 days after 2nd dose	Palpitation and sinus tachycardia	Stopped Thyroxine and started Carbimazole and propranolol.	Diagnosed with Graves' disease. Improvement in the thyroid function.
Pujol et al, 2022(3)	Case report	W, 38 years, schizophrenia	Pfizer/BioNtech BNT 16b2	12 days after 1st dose	Nervousness, insomnia and sweating	Methimazole	Diagnosed with Graves' disease
Taieb et al, 2022(58)	Case report	W, 43 years , no medical history	Pfizer/BioNtech BNT 16b2	3 days after 1st dose	Diarrhea and palpitations	Thiamazole and propranolol	Diagnosed with Graves' disease. TFTs normalised after 3 months
Chee et al, 2022(59)	Case series	W, 33 years	mRNA	7 days after 1st dose	NA	Carbimazole and propranolol	Diagnosed with Graves' disease. TFTs normalised after 28 days
		W, 37 years	mRNA	7 days after 1st dose	NA	Carbimazole and propranolol	Diagnosed with Graves' disease. TFTs normalised after 32 days
		W, 37 years	mRNA	21 days after 2st dose	NA	Carbimazole and propranolol	Diagnosed with Graves' disease. TFTs normalised after 53 days
		M, 34 years	mRNA	26 days after 1st dose	NA	Carbimazole and propranolol	Diagnosed with Graves' disease. TFTs normalised after 58 days
		W, 33 years	mRNA	9 days after 2st dose	NA	Carbimazole and propranolol	Diagnosed with Graves' disease. TFTs normalised after 64 days
		W, 43 years	mRNA	13 days after 2st dose	NA	Carbimazole	Diagnosed with Graves' disease. TFTs normalised after 57 days
		M, 59 years	mRNA	21 days after 1st dose	NA	Carbimazole	Diagnosed with Graves' disease. Still in follow up

		W, 74 years	mRNA	Asymptomatic (TFTs performed aspart of routine follow up 11 days after 2nd dose)	NA	Carbimazole	Diagnosed with Graves' disease.
		W, 25 years	mRNA	Asymptomatic (TFT performed as part of routine follow up 31 days after 2nd dose)	NA	Carbimazole	Diagnosed with Graves' disease. TFTs normalised after 123 days
		W, 41 years	mRNA	28 days after 2st dose	NA	Carbimazole	Diagnosed with Graves' disease. TFTs normalised after 31 days
		W, 24 years	mRNA	Asymptomatic (TFTs performed aspart of routine follow up 63 days after 2nd dose)	NA	Carbimazole	Diagnosed with Graves' disease. TFTs normalised after42 days
		W, 22 years	mRNA	5 days after 1st dose	NA	Carbimazole and propranolol	Diagnosed with Graves' disease. TFTs normalised after 178 days
Patrizio et al, 2022(60)	Case series	W, 58 years GD and GO in remission for 2 year (definitive therapy for GD with radioactive iodine)	Pfizer/BioNtechBNT 16b2	3 days after 2nd dose	Chemosis, redness of eyelids and conjunctiva, periorbital edema, painnand foreign object sensation innthe eyes and diplopia, CAS 6/10	Teprotumumab	Diagnosed with worsening of Graves' ophthalmopathy
		M, 43 years, previous GD 1 year before	Pfizer/BioNtechBNT 16b2	14 days after 1st dose	Proptosis, CAS 8/10, abduction deficit with diplopia, bilateral exposure keratopathy due to lagophthalmos	NA	Diagnosed with worsening of Graves' ophthalmopathy
Park et al, 2022(61)	Case series	W, 66 years, history of GD (treated with radioactive iodine) and TED stabile for over15 years	Moderna mRNA-1273	21 days after 2nd dose	Diplopia, bilateral eye-bulging, and pain with eye movement, CAS 6/10	Teprotumumab	Diagnosed with thyroid eye disease reactivation.Symptoms were improving at 5 months
		W, 53 years, recently started methimazole for low TSH	Pfizer/BioNtechBNT 16b2	1 day after 1st dose	Bulging and pain with eye movement in the right eye and occasional diplopia	Teprotumumab	Diagnosed with new onset of thyroid eye disease. Symptoms were improving at 8 months
		W, 45 years, history of Hashimoto's thyroiditis, TED, stable for 5 years, and bilateral hyaluronic acid fillerinjections several months before.	Moderna mRNA-1273	21 days after 1st dose, worsened afterthe 2nd	Worsening eyelid swelling, pain, redness, or diplopia.	No medication	Diagnosed with thyroid eye disease reactivation. Four months later, the eyelid swelling reduced, and her thyroid function tests normalised without treatment.
Hamouche etal, 2022(62)	Case report	M, 32 years, diagnosis of COVID-19 infection 10 days after 1st dose	Pfizer/BioNtechBNT 16b2	17 days after 1st dose	Palpitations, insomnia, tremor, irritability, diaphoresis, and dyspnoea at rest.	Methimazole, propranolol, 7-day course of Prednisone	Diagnosed with Graves' disease. After six weeks of treatment, the patient's symptoms improved progressively, and T3 and T4 levels normalised withintwo months.
Bostan et al, 2022(63)	Case series	W, 44 years, GD in remission (for 12 years)	Cororavac	7 days after 1st dose	Excessive sweating, palpitation, and fatigue, enlarged thyroid gland.	Methimazole andpropranolol	Diagnosed with relapsed Graves' Disease
		M, 49 years, GD in remission (for 1 year)	Pfizer/BioNtechBNT 16b2	30 days after 2nd dose	Palpitations, hand tremors, and sweating	Methimazole andpropranolol	Diagnosed with relapsed Graves' Disease. TFTsnormalised after 1 month

		W, 31 years GD in remission (for 1 year), breast cancer in remission.	Pfizer/BioNtechBNT 16b2	21 days after 1st dose	Hot flushes, weakness, and sweating	Methimazole andpropranolol	Diagnosed with relapsed Graves' Disease. TFTsnormalised after 5 weeks
		W, 51 years, DM, HT	Pfizer/BioNtechBNT 16b2	4 days after 2nd dose	Proptosis, irritation, and dryness, especially in the right eye, impaired blood pressure control,palpitations, sweating, and fever.	Methimazole and propranolol. Total thyroidectomy (after 4 monthof medical treatment)	Diagnosed with rapidly developing Graves' ophthalmopathy. After thyroidectomy, her ocularfindings showed a significant regression.
		W, 47 years, no medical history	Pfizer/BioNtech BNT 16b2	5 days after 1st dose	Sweating and palpitations	Methimazole and propranolol	Diagnosed with Graves' disease. TFTs normalised after 1 month
		W, 46 years, obesity	Pfizer/BioNtech BNT 16b2	21 days after 2nd dose	Emotional lability, sweating, palpitations, and weight loss	Methimazole and propranolol	Diagnosed with Graves' disease. TFTs normalised after 1 month
		W, 53 years, Hashimoto's thyroiditis (for 2 years)	Pfizer/BioNtechBNT 16b2	7 days after 2nddose	Palpitations, sweating, and weight loss	Stopped LT. Started Methimazole and propranolol	Conversion from Hashimoto's thyroiditis to Graves' disease. Two months later, the fT4 level decreased to 0.9 ng/dL and fT3 decreased to 2.37 ng/L, and the MMI dose was reduced to 10 mg/day.
Singh et al, 2022(64)	Case series	M, 42 years, no medical history	Moderna mRNA-1273	2 days after 3rd dose	Nausea, muscle weakness, sweating, weight loss, difficult in sleeping	Methimazole andpropranolol	NA
		F, 68 years, no medical history	Janssen Ad26.COV2.S	1 day after 1st dose	Constellation of non-specific symptoms and atrial fibrillation	Methimazole andbetablocker	NA
Chua, 2022(65)	Case series	M, 41 years, history ofGD in remission	Moderna mRNA-1273	1 day after 1st dose	Generalized muscle aches and weakness tremors and palpitations	Carbimazole	Diagnosed with relapsed Graves' Disease
		W, 45 years, no medical history	Pfizer/BioNtech BNT 16b2	2 days after 1st dose	Chest tightness and palpitations generalized body aches and fever	Carbimazole	Diagnosed with new onset Graves' disease
Manta et al, 2022(66)	Case report	M, 22 years, history of ulcerative colitis and nephrotic syndrome	Pfizer/BioNtech BNT 16b2	2 weeks after 1st dose	Subtle tremors in both upper extremities; TFTs consistent with hyperthyroidism, positivity of TRABs	Methimazole; subsequent hypothyroidism (treated)	Diagnosed with new onset Graves' disease; progression to hypothyroidism
Sakai et al, 2022(67)	Case report	W, 31 years, history of type 1 diabetes mellitus; previous silent thyroiditis at 23 years old	Pfizer/BioNtech BNT 16b2	7 days after 2nd dose	Excessive sweating, diarrhea, and shortness of breath during exertion; TFTs consistent with hyperthyroidism; ultrasonography revealed diffuse hyper perfusion in the thyroid gland	Methimazole	Diagnosed with new onset Graves' disease; thyroid function normalization after 3 months of therapy
Cuenca et al, 2022(68)	Case report	M, 57 years, not known medical history	VaxzevriaChAdOx1	2 days after vaccine	Tremor, palpitations, 3 kg weight loss, fatigue; TFTs consistent with hyperthyroidism; negative TRABs; thyroid ultrasound and scintigraphy consistent with Graves' disease	Thiamazole and propranolol	Diagnosed with new onset Graves' disease; thyroid function normalization after 6 months
Bres et al, 2022(42)	Case series	W, 44 years, known for Hashimoto's thyroiditis under levothyroxine	Pfizer/BioNtech BNT 16b2	5 days after 1st dose	Low TSH levels; thyroid ultrasound showing enlarged and hyper vascularized gland; positivity of TRABs	Reduction of levothyroxine	Diagnosed with conversion from Hashimoto's to Graves' disease; received 2nd dose without further complications
Ruggeri et al, 2022(69)	Case series	M, 50 years, no significant medical history	Pfizer/BioNtech BNT 16b2	14 days after 1st dose	Fatigue, palpitations, distal tremor, insomnia, anxiety, and irritability; TFTs consistent with hyperthyroidism, thyroid ultrasound and scintigraphy suggestive for Graves' disease	Methimazole	Diagnosed with new onset Graves' disease; thyroid function normalization after 6 months (under low dose of methimazole)

Other thyroid function alterations

Author, year	Study design	Patient(s)	Type of vaccine	Latency between vaccine and symptoms	Clinical presentation	Treatment	Outcome/Conclusions
Giusti et al, 2021(70)	Case report	W, 61 years, nomedical history	Pfizer/BioNtechBNT 16b2	21 days after 2nd dose	Swelling of neck and face,without pain, asthenia, andweight gain	Levothyroxine and Betamethasone	Diagnosed with overt hypothyroidism and myxoedema features. TFTs normalised after 3 months. Decrease of thyroid volume. Positive thyroid antibodies and the hypoechoic pattern of the gland slightly changed. The neck and facial swelling of the patient improved, while her body weight only minimallychanged.
Raven et al, 2022(47)	Case report	W, 35 years, previous history of left hemithyroidectomy 3 years prior for a benign 31-mm thyroid nodule	Pfizer/BioNtechBNT 16b2	4 days after 1st dose	Right side neck pain. Thyroid function remained normal.	No treatment	Diagnosed with focal painful Thyroiditis After 2nd dose vaccine, neck pain increased along with fatigue, fevers, and night sweats. Over the following 2 weeks, the pain resolved.
Capezzone et al, 2022(71)	Case series	M, 34 years, no medical history	Moderna mRNA-1273	7 days after 1st dose	Palpitation and weight loss	No treatment	Diagnosed with silent thyroiditis. TFTs normalised after 33 days
		W, 29 years, no medical history	Moderna mRNA-1273	7 days after 1st dose	Palpitation and weight loss	No treatment	Diagnosed with silent thyroiditis. TFTs normalized after 29 days
Nakaizumi et al, 2022(72)	Case series	W, 38 years	Pfizer/BioNtech BNT 16b2	17 days after 1st dose	Palpitations	No treatment	Diagnosed with focal painless thyroiditis. TFTs normalised after 5 months
		W, 59 years	Pfizer/BioNtechBNT 16b2	10 days after 2nd dose	Abnormal TFTs was incidentally detected on a blood test. She was asymptomatic, and her physical examination results were unremarkable.	No treatment	Diagnosed with focal painless thyroiditis. TFTsnormalised after 2 months
Yamamoto etal, 2022(73)	Case report	W, 64 years, history of colorectal cancer, diabetes mellitus, obesity	Pfizer/BioNtechBNT 16b2	4 days after 1st dose	Shortness of breath palpitations, worsening respiratory distress, decreased urine output, edema of both lower legs, and a fever, heartfailure complicated with atrial fibrillation	Thiamazole, potassium iodine, corticosteroid, furosemide and Carvedilol	She was diagnosed as having a thyrotoxic crisis complicated with atrial fibrillation, and heart failure. General condition improved rapidly, and her respiratory distress disappeared after 5 days. TFTs normalized after2 weeks. The patient refused thyroidectomy and preferred to be maintained by medication.
Ruggeri et al, 2022(69)	Case series	W, 55 years, known for Hashimoto's thyroiditis, euthyroid	Moderna mRNA-1273	10 days after 1st dose	Fatigue, tremors and palpitations. TFTs demonstrated suppressed TSH, elevated FT4 and FT3 levels; scintigraphy consistent with destructive thyroiditis	No treatment	Four weeks later, her TFTs showed subclinical hypothyroidism. Six months after, due to persistent subclinical hypothyroidism, levothyroxine treatment was started, with subsequent TFTs normalization
		M, 61 years, previous history of Graves' disease, in remission	Pfizer/BioNtechBNT 16b2	15 days after 3rd dose	Fatigue and muscle weakness, decreased concentration, loss of memory, depression, increased sensitivity to cold, constipation. TFTs revealed overt hypothyroidism with TPOAb positivity	Levothyroxine	Diagnosed with autoimmune hypothyroidism. TFTs normalization at follow-up after 6 months
Marsukjai et al, 2022(74)	Case report	W, 55 years, hypertension and hyper-cholesterolemia	Vaxzevria ChAdOx1	14 days after 2nd dose	Palpitations. High troponin I values and TFTs suggestive for hyperthyroidism; thyroid ultrasound suggestive for SAT; cardiac MRI: myocarditis	Propranolol	Concomitant myocarditis and painless thyroiditis. Spontaneous normalization of thyroid function
Poli et al, 2022(75)	Case report	M, 65 years, with a medical history of acute disseminated encephalomyelit	Pfizer/BioNtech BNT 16b2	3 days after 3rd dose	Paresis of the left arm, loss of pain and temperature sensation on the right side of the body, right-sided deafness with vertigo, fluctuating binocular horizontal diplopia and	high-dose intravenous methylprednisolone, intravenous immunoglobulin therapy, plasmapheresis, pyrydostigmine	Diagnosed with myasthenia gravis and autoimmune thyroiditis. After plasmapheresis, anti-thyroid antibodies turned out negative

		is			ptosis of the right eye; Immunologic testing showed elevated anti-acetylcholine antibody, anti-thyroglobulin antibodies, anti-thyroid peroxidase antibodies and anti-TSH receptor antibodies positivity (Thyroid function was normal)		

Adrenal gland

Author, year	Study design	Patient(s)	Type of vaccine	Latency between vaccine and symptoms	Clinical presentation	Treatment	Outcome/Conclusions
Taylor et al, 2021(76)	Case report	M, 38 years, no significant medical history	Vaxzevria ChAdOx1	8 days after 1st dose	Severe abdominal pain and vomiting. Bilateral adrenal haemorrhage (CT findings); pulmonary embolism, cerebral venous thrombosis. Exams consistent with VITT	Intravenous hydrocortisone and methylprednisolone, intravenous immunoglobulins and direct thrombin inhibitor; plasma exchange	Diagnosed with bilateral adrenal haemorrhage. Discharged at home with chronic steroid therapy on the presumption of long-term primary adrenal insufficiency
Varona et al, 2021(77)	Case report	M, 47 years, no significant medical history	Vaxzevria ChAdOx1	10 days (not specified after which dose)	Headache, somnolence, hypotension, abdominal pain. Bilateral pulmonary embolism, cerebral venous thrombosis, bilateral adrenal haemorrhage (MRI findings); low cortisol, exams consistent with VITT	Hormone replacement therapy with hydrocortisone, intravenous immunoglobulins and subcutaneous fondaparinux	Diagnosed with bilateral adrenal haemorrhage. Discharged at home with chronic steroid therapy on the presumption of long-term primary adrenal insufficiency
Tews et al, 2022(78)	Case report	M, 39 years, no significant medical history, active smoker	Janssen Ad26.COV2.S	8 days after 1st dose	Severe pain in the left thorax and upper abdomen. Deep vein thrombosis, bilateral pulmonary embolism, bilateral adrenal haemorrhage (MRI findings). Exams consistent with VITT	Hormone replacement therapy with hydrocortisone, intravenous immunoglobulins and direct thrombin inhibitor	Diagnosed with bilateral adrenal haemorrhage. Discharged at home with chronic steroid therapy on the presumption of long-term primary adrenal insufficiency
Blauenfeldt et al, 2021(79)	Case report	W, 60 years, Hashimoto thyroiditis and hypertension	Vaxzevria ChAdOx1	7 days after 1st dose	Intractable abdominal pain, left-side signs. Bilateral adrenal haemorrhage (CT scan findings), massive ischemic stroke (occlusion of right internal carotid artery). Thrombocytopaenia and positivity to anti PF-4 antibodies	Hormone replacement therapy with hydrocortisone, platelet transfusion, hemicraniectomy, dalteparin, palliative treatment	Diagnosed with acute ischemic stroke and bleeding. Deceased after 6 days of hospitalization
D'Agostino et al, 2021(80)	Case report	W, 54 years, Meniere's disease	Vaxzevria ChAdOx1	12 days after 1st dose	Left-side signs; signs of DIC. Multiple subacute cerebral hemorrhages, acute basilar thrombosis and superior sagittal sinus thrombosis, myocardial infarction; right suprahepatic vein thrombosis, bilateral pulmonary embolism, bilateral adrenal haemorrhage (CT scan findings). Thrombocytopenia	Angioplasty, intensive care treatment	Diagnosed with DIC and cerebral venous thrombosis. Deceased after 5 days of hospitalization
Maguire et al, 2021(81)	Case-series	5 (3W, 2M) aged 41-74 years, with known adrenal insufficiency	Vaxzevria ChAdOx1	8-24 h after 1st dose	Headache, severe fatigue, abdominal cramps, watery diarrhoea, nausea	Double dose of oral steroid for 2 days/ intramuscular steroid	Diagnosed with adrenal crisis. Rapidly improved

Haji et al, 2021(82)	Case report	M, 63 years, no significant medical history, daily marijuana use	Janssen Ad26.COV2.S	24 h after 1st dose	Neuroendocrine crisis with nausea, vomiting, dyspnoea, watery diarrhoea, chills, sweats, and heavy chest pain, acute hypertension. Elevated 24h urinary metanephrines; 7 cm heterogenic mass in right adrenal gland (ultrasound)	Intubated, antihypertensive drugs, adrenal mass resection (pheochromocytoma)	Diagnosed with pheochromocytoma. Discharged at home with chronic therapy and endocrinologic follow-up indication
Al Rawahi et al, 2021(83)	Case report	M, 64 years, hypertension and hyperlipidemia	Vaxzevria ChAdOx1	7 days (not specified after which dose)	Fever, lethargy, abdominal pain. Pulmonary embolism, left renal vein thrombosis with renal infarction, bilateral adrenal haemorrhage (CT scan findings). exams consistent with VITT	Hormone replacement therapy with hydrocortisone, platelet transfusion, argatroban and fondaparinux	Diagnosed with bilateral adrenal haemorrhage. Discharged at home with chronic steroid therapy
Graf et al, 2022(84)	Case series	1M, 46 years, current smoker and drinker 1W, 38 years, gastro-oesophageal reflux disease, osteoarthritis, obesity	Vaxzevria ChAdOx1	8-11 days after 1st dose	Abdominal and back pain, nausea and vomiting; pulmonary embolism, portal vein thrombosis, renal cortical infarcts, myocardial infarction, dural venous sinus thrombosis, cerebral infarction, bilateral/ unilateral adrenal haemorrhage (CT scan findings); exams consistent with VITT	Hormone replacement therapy with hydrocortisone, intensive care treatment	Diagnosed with bilateral/ unilateral adrenal haemorrhage. Discharged at home with chronic steroid therapy
Efthymiadis et al, 2022(85)	Case report	W, 23 years, obesity, family history (not 1st degree) of venous thromboembolism	Vaxzevria ChAdOx1	8 days after 1st dose	Acute pleuritic chest pain, fever and headaches, seizures. CT and MRI findings (at 16 days after 1st dose): multiple lobar and segmental pulmonary emboli, bilateral adrenal haemorrhage and splenic vein thrombosis, posterior reversible encephalopathy Exams consistent with VITT, positive anti-PF4 antibodies	Intensive care treatment, anticoagulants, intravenous hydrocortisone	Diagnosed with bilateral adrenal haemorrhage. Discharged at home with chronic steroid and anticoagulant therapy
Markovic et al, 2022(86)	Case report	M, 74 years, known for adrenal insufficiency (hypopituitarism), T2DM, hypertension	Pfizer/BioNtech BNT 16b2	Few hours after the 2nd dose	Altered mental status and fever, severe hypoglicaemia	Intravenous hydro-cortisone	Diagnosed with adrenal crisis. Rapidly improved

Pituitary Gland

Author, year	Study design	Patient(s)	Type of vaccine	Latency between vaccine and symptoms	Clinical presentation	Treatment	Outcome/Conclusions
Murvelashvili et al, 2021(87)	Case report	M, 51 years, no significant medical history	Moderna mRNA-1273	3 days after 1st dose; 2 days after 2nd dose	Nausea, vomiting, abdominal pain; blood examinations showed hyponatremia, central hypothyroidism, secondary hypogonadism, undetectable cortisol; enlargement of the pituitary gland (CT and MRI findings) and thickening of the pituitary stalk (MRI)	Intravenous dexamethasone and thyroid hormone replacement	Diagnosed with hypophysitis. after 1 month, MRI revealed markedly diminished enlargement with a mostly empty sella

Lindner et al, 2021(88)	Case report	M, 79 years, previous ischemic cerebrovascular insult, gastritis and gastroesophageal refux	Moderna mRNA-1273	10 days after 2nd dose	Weakness, fatigue and anorexia. Hyponatremia with high urine osmolality	Intravenous crystalloid solutions, fluid restriction, oral urea	Diagnosed with Syndrome of Inappropriate Antidiuresis (SIADH)
Pinar-Gutiérrez et al, 2022(89)	Case report	W, 37 years, not known medical history	Vaxzevria ChAdOx1	5 days after 1st dose	High-intensity frontal headache: at MRI, adenohypophysis haemorrhagic bleeding in association with a 10 mm intraglandular adenoma without chiasmatic involvement was found; no hormonal deficits	None	Diagnosed with pituitary apoplexy without hormonal deficits
Roncati et al, 2022(90)	Case report	W, 28 years, no significant medical history	Vaxzevria ChAdOx1	24h after the 1st dose - 2 months	Tension type headache worsened after second dose, accompanied by amenorrhea and hyperprolactinemia; MRI performed 69 days after second dose showed haemorrhagic event in the right half of the sella	Not reported	Diagnosed with pituitary apoplexy with secondary amenorrhea
Bouça et al, 2022(91)	Case report	W, 37 years, rheumatoid arthritis	Pfizer/BioNtech BNT 16b2	7 days after the 2nd dose	Intense thirst and polyuria, urine volume 10200 mL/24h, urine osmolality 75 mOs/Kg, urine density 1.002; MRI showed loss of the posterior pituitary bright spot	Oral desmopressin 0.06 mg twice a day	Diagnosed with Central Diabetes Insipidus
Ankireddypalli et al, 2022(92)	Case report	W, 48 years, no significant medical history	Pfizer/BioNtech BNT 16b2	2 days after the 1st dose	Flu-like symptoms, severe headache, myalgia, polyuria (4l/die), polydipsia, fatigue, nausea, weight loss, low IGF-1, no menses after the 1st dose; MRI revealed a 4 mm, round, thickened pituitary stalk and a partially empty sella	10 mcg of desmopressin nasal sprays twice a day	Diagnosed with hypophysitis with acute onset of diabetes insipidus
Morita et al, 2022(93)	Case report	M, 31 years	Pfizer/BioNtech BNT 16b2	1-4 days after the 2 nd dose	General fatigue, fever, headache, nausea, and diarrhoea; hyponatremia, hypoglycaemia, low plasma ACTH and serum cortisol levels; MRI (after the replacement with HC) revealed moderate atrophy of the pituitary gland with a preserved high intensity of the posterior pituitary gland by the T1-weighted image	Hydrocortisone replacement therapy	Diagnosed with isolated adrenocorticotropic hormone (ACTH) deficiency debuted with adrenal crisis
Mizuno et al, 2022(94)	Case report	M, 48 years, epilepsy (under valproate and levetiracetam), end-stage renal disease, smoker	Pfizer/BioNtech BNT 16b2	7 days after the 1st dose	fever, generalized weakness, diaphoresis, muscle rigid-ity, altered mental status. Blood examinations showed elevated creatine kinase (CK) and C-reactive protein (CRP), low ACTH and cortisol levels, confirmed at ACTH stimulation test	Intravenous followed by oral hydrocortisone	Diagnosed with neuroleptic malignant syndrome (NMS) with central adrenal insufficiency; discharged at home on oral hydrocortisone

Type 1 Diabetes Mellitus

New onset of T1DM

Author, year	Study design	Patient(s)	Type of vaccine	Latency between vaccine and symptoms	Clinical presentation	Treatment	Outcome/Conclusions
Patrizio et al, 2021(53)	Case report	M, 52 years, type 2 diabetic treated with oral antidiabetic agents, vitiligo vulgaris	Pfizer/BioNtech BNT 16b2	28 days after 2nd dose	Fever, weight loss, asthenia, hyperthyroidism, poor glycaemic control; positivity of thyrotropin receptor antibodies and thyroid enlargement; positivity of pancreatic autoantibodies against glutamic acid decarboxylase 65, very low serum C-peptide level	Methimazole, Atenolol, Insulin	Diagnosed with acute onset type 1 diabetes and Graves' disease<br>Discharged at home with methimazole and Insulin therapy
Ohuchi et al, 2021(95)	Case report	M, 45 years, treated with nivolumab for melanoma	Pfizer/BioNtech BNT 16b2	3 days after 2nd dose	Fatigue, dry mouth, increased urine frequency, and prominent weight loss; hyperglycaemia, decreased C-peptide reactivity	Not reported	Diagnosed with fulminant type I diabetes mellitus caused by nivolumab, probably triggered by vaccination for COVID-19
Yano et al, 2022(96)	Case report	W, 51 years, no significant medical history	Moderna mRNA-1273	28 days after 1st dose	Fatigue, thirst, polyuria, polydipsia, weight loss, tachycardia and reduced skin turgor, suggesting mild dehydration; hyperglycaemia, ketoacidosis, exhaustion of endogenous insulin secretion, positive insulin autoantibody, positivity of HLA for type 1 diabetes. Symptoms significantly worsened 2 days after 2nd dose.	Intravenous fluids and insulin	Diagnosed with acute-onset type 1 diabetes; discharged at home with insulin
Tang et al, 2022(97)	Case report	M, 50 years, no significant medical history	Sinovac Biotech Coronavac	5 days after 1st dose	Fever, polydipsia, polyuria; hyperglycaemia, ketoacidosis, undetectable/low serum C-peptide level, positivity of HLA for fulminant type 1 diabetes	Intravenous fluids and insulin	Diagnosed with fulminant type 1 diabetes. DKA was resolved promptly, and he was placed on a subcutaneous insulin regimen. Four weeks after disease onset, he had an almost complete loss of islet function
Sasaki et al, 2022(98)	Case report	W, 45 years<br>Bronchial asthma	Pfizer/BioNtech BNT 16b2	6 days after 2nd dose	Nausea, abdominal pain, fatigue, polydipsia and weight loss; hyperglycaemia, ketoacidosis, exhaustion of endogenous insulin secretion, positivity of HLA for fulminant type 1 diabetes	Intravenous fluids and insulin	Diagnosed with fulminant type 1 diabetes. Discharged at home with insulin
Sasaki et al, 2022(99)	Case report	W, 73 years, known for glucose intolerance treated with diet and exercise	Moderna mRNA-1273	49 days after 2nd dose	Anorexia, fatigue, nausea and vomiting; hyperglycaemia, low serum C-peptide level, positivity of anti-glutamic acid decarboxylase antibody and insulin autoantibody, positivity of HLA for type 1 diabetes	Intensive insulin therapy	Diagnosed with type 1 diabetes without complications
Sakurai et al, 2022(100)	Case report	W, 36 years, no significant medical history	Pfizer/BioNtech BNT 16b2	3 days after 1st dose	Thirst, polydipsia, polyuria, palpitations, loss of appetite, fatigue; hyperglycaemia, ketoacidosis, decrease serum C-peptide, positivity of HLA for type 1 diabetes	Intravenous fluids and insulin	Diagnosed with acute-onset type 1 diabetes; discharged at home with insulin

Makiguchi et al, 2022(101)	Case report	W, 65 years, lung carcinoma with brain metastases, panhypopituitarism, under immuno-therapy	Pfizer/BioNtech BNT 16b2	A few days after 2nd dose	Fatigue, extensive erythema on the trunk; ketoacidosis, markedly decreased of urine C-peptide level	Unknown	Diagnosed with acute-onset type 1 diabetes
Bleve et al, 2022(102)	Case series	W, 57 years, family history for T2D and autoimmune diseases (vitiligo and Hashimoto thyroiditis)	Vaxzevria ChAdOx1-S	A few days after 1st dose	Polydipsia, elevated blood glucose values (> 300 mg/dL), glycosuria (> 1000 mg/dL), ketonuria and asthenia; elevated levels of anti-GAD, Anti-IA2, and antitransglutaminase IgA antibody were found	Intravenous fluids and insulin	Diagnosed with autoimmune diabetes
		W, 61 years, hypothyroidism	Pfizer/BioNtech BNT 16b2	A few days after 2nd dose	Dyspnoea, nausea, and abdominal pain, polyuria, polydipsia, and asthenia; elevated levels of FPG (640 mg/dl) and metabolic acidosis; elevated levels of anti-GAD	Intravenous fluids and insulin	Diagnosed with autoimmune diabetes. Discharged with basal-bolus insulin therapy regimen
Aydoğan et al, 2022(103)	Case series	M, 56 years, vitiligo, Hashimoto's thyroiditis	Pfizer/BioNtech BNT 16b2	15 days after the 2nd dose	Weight loss, dry mouth, polyuria, and polydipsia; high levels of FPG; positive anti-GAD65	Intravenous fluids and insulin	Discharged insulin therapy; discontinued treatment after 3 months due to hypoglycaemia
		M, 48 years, no significant medical history	Pfizer/BioNtech BNT 16b2	2 months after the 2nd dose	Fatigue, high FPG and HbA1c, positive anti-GAD65	The patient refused insulin treatment, but received medical nutrition therapy	Nutrition therapy
		M, 36 years, no significant medical history	Sinovac Biotech Coronavac (2 doses) Pfizer/BioNtech BNT 16b2 (2 doses)	1 month after 2nd Pfizer/BioNtech dose	Fatigue, dizziness, unintentional weight loss, and dry mouth; elevated levels of FPG (656 mg/dl) and metabolic acidosis; elevated levels of anti-GAD	Intravenous fluids and insulin	Diagnosed with autoimmune diabetes. Discharged with basal-bolus insulin therapy regimen
		W, 27 years, no significant medical history	Pfizer/BioNtech BNT 16b2	3 weeks after the 2nd dose	Blurred vision, polyuria, polydipsia, weight loss, and vaginal candidiasis, high FPG and HbA1c, positive anti-GAD65	Basal-bolus insulin therapy regimen	Diagnosed with autoimmune diabetes. Discharged with basal-bolus insulin therapy regimen; discontinued
Sato et al, 2022(104)	Case report	M, 43 years, malignant melanoma under nivolumab	mRNA-based SARS-CoV-2 vaccination	2 days after the 2nd dose	Thirst, polydipsia, and polyuria, weight loss, elevated levels of FPG and metabolic acidosis; panel for autoimmunity tested negative	Intravenous fluids and insulin	Discharged with basal-bolus insulin therapy regimen

Glycaemic alterations in T1DM Patients

**Author, year**	**Study design**	**Patient(s)**	**Type of vaccine**	**Latency between vaccine and symptoms**	**Clinical presentation**	**Treatment**	**Outcome/Conclusions**
Yakou et al, 2022(105)	Case series	W, 71 years, type 1 diabetes mellitus at 56 years of age, history of Basedow disease	Pfizer/BioNtech BNT 16b2	Less than 12 h after 1st dose	Nausea, fatigue, impaired consciousness, tachycardia and tachypnea; severe ketoacidosis, undetectable serum C-peptide level, positive for antiglutamic acid decarboxylase antibody	Intravenous insulin, Ringer's solution, and glucose infusion	Diagnosed with DKA. Discharged with home therapy
		W, 52 years, poorly controlled type 1 diabetes mellitus for 5 years, drinking habit - consumed 20 g of alcohol on the night before vaccine	Pfizer/BioNtech BNT 16b2	Less than 12 h after 2nd dose	Nausea, palpitation, respiratory distress, tachycardia and tachypnea; hyperglycaemia, ketoacidosis	Intravenous insulin, Ringer's solution, and glucose infusion	Diagnosed with DKA. Discharged with home therapy

Ganakumar et al, 2022(106)	Case series	M, 20 years, type 1 diabetes mellitus for 6 years	Vaxzevria ChAdOx1	3 days after 2nd dose	Abdominal pain, nausea, vomiting; severe ketoacidosis	Intravenous fluids and insulin	Diagnosed with severe DKA. Discharged with home therapy
		W, 25 years, type 1 diabetes mellitus for 6 years	Bharat Biotech BBV512	6 days after 2nd dose	Fever, myalgia, nausea, vomiting, abdominal pain; severe ketoacidosis	Intravenous fluids and insulin	Diagnosed with severe DKA. Discharged with home therapy
Zilbermint et al, 2022(107)	Case report	W, 24 years, known for T1DM	Moderna mRNA-1273	15 hours after 2nd dose	Severe nausea, tachycardia, tachypnoea; hyperglycaemia, ketoacidosis, transient insulin resistance	Intravenous fluids and insulin	Diagnosed with DKA. Discharged with home therapy
Infante et al, 2022(108)	Case report	M, 24 years, known for T1DM under only low basal insulin daily doses	Pfizer/BioNtech BNT 16b2	First days after both doses	Deterioration in his glucose control, with moderate increase in the frequency of postprandial hyperglycemic episodes	Need to resume the mealtime rapid-acting insulin injections for 7 days	Three months after vaccines shots the patient was still in the honeymoon phase

Male reproductive system

Author, year	Study design	Patient(s)	Type of vaccine	Latency between vaccine and symptoms	Clinical presentation	Treatment	Outcome/Conclusions
Chatzimeletiou et al, 2022(109)	Case report	M, 43 years with ankylosing spondylitis	Pfizer/BioNtech BNT 16b2	/	Semen analysis performed during therapy with anti-inflammatory drugs before and after 3 doses of vaccine	/	No negative effect on sperm parameters

Abbreviations: SAT: subacute thyroiditis; TFTs: thyroid function tests; NSAIDs: Non-steroidal anti-inflammatory drugs; NA: not acquired; TPOAb: thyroid peroxidase antibodies; TRAbs: TSH receptor antibodies; CT: computed tomography; VITT: Vaccine-Induced Immune Thrombotic Thrombocytopenia; PF-4: platelet factor 4; MRI: magnetic resonance imaging; DIC: disseminated intravascular coagulation; SIADH: Syndrome of Inappropriate Antidiuresis; ACTH: adrenocorticotropic hormone; T1DM: type 1 diabetes mellitus; DKA: diabetic ketoacidosis; HLA: Human Leukocyte Antigen; GAD: glutamic acid decarboxylase; IA2: Islet Antigen 2; FPG: fasting plasma glucose; T2DM: type 2 diabetes mellitus

1. İremli BG, Şendur SN, Ünlütürk U. Three Cases of Subacute Thyroiditis Following SARS-CoV-2 Vaccine: Postvaccination ASIA Syndrome. J Clin Endocrinol Metab. 2021;106(9):2600-5.
2. Franquemont S, Galvez J. Subacute Thyroiditis After mRNA vaccine for Covid-19. J Endocr Soc. 2021;5:A956-A7.
3. Pujol A, Gómez LA, Gallegos C, Nicolau J, Sanchís P, González-Freire M, et al. Thyroid as a target of adjuvant autoimmunity/inflammatory syndrome due to mRNA-based SARS-CoV2 vaccination: from Graves' disease to silent thyroiditis. J Endocrinol Invest. 2022;45(4):875-82.
4. Saygılı ES, Karakilic E. Subacute thyroiditis after inactive SARS-CoV-2 vaccine. BMJ Case Rep. 2021;14(10).
5. Şahin Tekin M, Şaylısoy S, Yorulmaz G. Subacute thyroiditis following COVID-19 vaccination in a 67-year-old male patient: a case report. Hum Vaccin Immunother. 2021;17(11):4090-2.
6. Bornemann C, Woyk K, Bouter C. Case Report: Two Cases of Subacute Thyroiditis Following SARS-CoV-2 Vaccination. Front Med (Lausanne). 2021;8:737142.
7. Plaza-Enriquez L, Khatiwada P, Sanchez-Valenzuela M, Sikha A. A Case Report of Subacute Thyroiditis following mRNA COVID-19 Vaccine. Case Rep Endocrinol. 2021;2021:8952048.
8. Siolos A, Gartzonika K, Tigas S. Thyroiditis following vaccination against COVID-19: Report of two cases and review of the literature. Metabol Open. 2021;12:100136.
9. Schimmel J, Alba EL, Chen A, Russell M, Srinath R. Thyroiditis and Thyrotoxicosis After the SARS-CoV-2 mRNA Vaccine. Thyroid. 2021;31(9):1440.
10. Oyibo SO. Subacute Thyroiditis After Receiving the Adenovirus-Vectored Vaccine for Coronavirus Disease (COVID-19). Cureus. 2021;13(6):e16045.
11. Ratnayake GM, Dworakowska D, Grossman AB. Can COVID-19 immunisation cause subacute thyroiditis? Clin Endocrinol (Oxf). 2021.
12. Chatzi S, Karampela A, Spiliopoulou C, Boutzios G. Subacute thyroiditis after SARS-CoV-2 vaccination: a report of two sisters and summary of the literature. Hormones (Athens). 2022;21(1):177-9.
13. Jeeyavudeen MS, Patrick AW, Gibb FW, Dover AR. COVID-19 vaccine-associated subacute thyroiditis: an unusual suspect for de Quervain's thyroiditis. BMJ Case Rep. 2021;14(11).
14. Kyriacou A, Ioakim S, Syed AA. COVID-19 vaccination and a severe pain in the neck. Eur J Intern Med. 2021;94:95-6.
15. Soltanpoor P, Norouzi G. Subacute thyroiditis following COVID-19 vaccination. Clin Case Rep. 2021;9(10):e04812.
16. Lee KA, Kim YJ, Jin HY. Thyrotoxicosis after COVID-19 vaccination: seven case reports and a literature review. Endocrine. 2021;74(3):470-2.
17. Khan F, Brassill MJ. Subacute thyroiditis post-Pfizer-BioNTech mRNA vaccination for COVID-19. Endocrinol Diabetes Metab Case Rep. 2021;2021.
18. Leber HM, Sant'Ana L, Konichi da Silva NR, Raio MC, Mazzeo TJMM, Endo CM, et al. Acute Thyroiditis and Bilateral Optic Neuritis following SARS-CoV-2 Vaccination with CoronaVac: A Case Report. Ocul Immunol Inflamm. 2021;29(6):1200-6.
19. Sözen M, Topaloğlu Ö, Çetinarslan B, Selek A, Cantürk Z, Gezer E, et al. COVID-19 mRNA vaccine may trigger subacute thyroiditis. Hum Vaccin Immunother. 2021;17(12):5120-5.
20. Pandya M, Thota G, Wang X, Luo H. Thyroiditis after Coronavirus Disease 2019 (COVID-19) mRNA Vaccine: A Case Series. AACE Clin Case Rep. 2021.
21. Sigstad E, Grøholt KK, Westerheim O. Subacute thyroiditis after vaccination against SARS-CoV-2. Tidsskr Nor Laegeforen. 2021;141(2021-14).
22. González López J, Martín Niño I, Arana Molina C. Subacute thyroiditis after SARS-CoV-2 vaccination: report of two clinical cases. Med Clin (Barc). 2021.
23. Rebollar AF. [SUBACUTE THYROIDITIS AFTER ANTI SARS-COV-2 (Ad5-nCoV) VACCINE]. Enferm Infecc Microbiol Clin. 2021.
24. Ippolito S, Gallo D, Rossini A, Patera B, Lanzo N, Fazzino GFM, et al. SARS-CoV-2 vaccine-associated subacute thyroiditis: insights from a systematic review. J Endocrinol Invest. 2022.
25. Yorulmaz G, Sahin Tekin M. SARS-CoV-2 vaccine-associated subacute thyroiditis. J Endocrinol Invest. 2022.

26. Patel KR, Cunnane ME, Deschler DG. SARS-CoV-2 vaccine-induced subacute thyroiditis. Am J Otolaryngol. 2022;43(1):103211.
27. Pla Peris B, Merchante Alfaro A, Maravall Royo FJ, Abellán Galiana P, Pérez Naranjo S, González Boillos M. Thyrotoxicosis following SARS-COV-2 vaccination: a case series and discussion. J Endocrinol Invest. 2022;45(5):1071-7.
28. Bostan H, Unsal IO, Kizilgul M, Gul U, Sencar ME, Ucan B, et al. Two cases of subacute thyroiditis after different types of SARS-CoV-2 vaccination. Arch Endocrinol Metab. 2022;66(1):97-103.
29. Jhon M, Lee SH, Oh TH, Kang HC. Subacute Thyroiditis After Receiving the mRNA COVID-19 Vaccine (Moderna): The First Case Report and Literature Review in Korea. J Korean Med Sci. 2022;37(6):e39.
30. Vasileiou V, Paschou SA, Tzamali X, Mitropoulou M, Kanouta F, Psaltopoulou T, et al. Recurring subacute thyroiditis after SARS-CoV-2 mRNA vaccine: A case report. Case Rep Womens Health. 2022;33:e00378.
31. Das L, Bhadada SK, Sood A. Post-COVID-vaccine autoimmune/inflammatory syndrome in response to adjuvants (ASIA syndrome) manifesting as subacute thyroiditis. J Endocrinol Invest. 2022;45(2):465-7.
32. Bahçecioğlu AB, Karahan ZC, Aydoğan BI, Kalkan IA, Azap A, Erdoğan MF. Subacute thyroiditis during the COVID-19 pandemic: a prospective study. J Endocrinol Invest. 2022;45(4):865-74.
33. Pipitone G, Rindi LV, Petrosillo N, Foti NAM, Caci G, Iaria C, et al. Vaccine-Induced Subacute Thyroiditis (De Quervain's) after mRNA Vaccine against SARS-CoV-2: A Case Report and Systematic Review. Infect Dis Rep. 2022;14(1):142-54.
34. Stasiak M, Zawadzka-Starczewska K, Lewiński A. Significance of HLA Haplotypes in Two Patients with Subacute Thyroiditis Triggered by mRNA-Based COVID-19 Vaccine. Vaccines (Basel). 2022;10(2).
35. Bennet WM, Elamin A, Newell-Price JD. Subacute thyroiditis following COVID-19 vaccination: Case report and Society for Endocrinology survey. Clin Endocrinol (Oxf). 2022.
36. Kishimoto M, Ishikawa T, Odawara M. Subacute thyroiditis with liver dysfunction following coronavirus disease 2019 (COVID-19) vaccination: report of two cases and a literature review. Endocr J. 2022.
37. Huo J, Qu R, Guo Y, Ou D. Nerve block therapy for subacute thyroiditis following COVID-19 vaccination: The first case report. Asian J Surg. 2022.
38. Adelmeyer J, Goebel JN, Kauka A, Kann PH. Two Case Reports of Subacute Thyroiditis after Receiving Vaccine for COVID-19. Case Rep Endocrinol. 2022;2022:3180004.
39. Borges Canha M, Neves JS, Oliveira AI, Sarmento A, Carvalho D. Subacute Thyroiditis After Severe Acute Respiratory Syndrome Coronavirus 2 Vaxzevria Vaccination in a Patient With Thyroid Autoimmunity. Cureus. 2022;14(2):e22353.
40. Oğuz SH, Şendur SN, İremli BG, Gürlek A, Erbas T, Ünlütürk U. SARS-CoV-2 Vaccine-induced Thyroiditis: Safety of Revaccinations and Clinical Follow-up. J Clin Endocrinol Metab. 2022;107(5):e1823-e34.
41. Topaloglu O, Tekin S, Topaloglu S, Bayraktaroglu T. Persistent Subacute Thyroiditis Post SARS-CoV-2 Vaccine in a Male Patient with Positive Thyroid Autoantibodies. TURKISH JOURNAL OF ENDOCRINOLOGY AND METABOLISM2022. p. 48-54.
42. Brès F, Joyeux MA, Delemer B, Vitellius G, Barraud S. Three cases of thyroiditis after COVID-19 RNA-vaccine. Ann Endocrinol (Paris). 2022;83(4):262-4.
43. Vu TB, Chu DT, Le DT, Hoang TTD, Gautret P, Hoang VT. Subacute thyroiditis after receiving the vaccine for COVID-19: a case report and literature review. Clin Exp Vaccine Res. 2022;11(2):226-9.
44. Murashita M, Wada N, Baba S, Sugawara H, Miyoshi A, Obara S. Subacute thyroiditis associated with thyrotoxic periodic paralysis after COVID-19 vaccination: a case report. Endocrinol Diabetes Metab Case Rep. 2022;2022.
45. Pi L, Lin J, Zheng Y, Wang Z, Zhou Z. Case Report: Subacute thyroiditis after receiving inactivated SARS-CoV-2 vaccine (BBIBP-CorV). Front Med (Lausanne). 2022;9:918721.
46. Zettinig G, Krebs M. Two further cases of Graves' disease following SARS-Cov-2 vaccination. J Endocrinol Invest. 2022;45(1):227-8.
47. Raven LM, McCormack AI, Greenfield JR. Letter to the Editor From Raven et al: "Three Cases of Subacute Thyroiditis Following SARS-CoV-2 Vaccine". J Clin Endocrinol Metab. 2022;107(4):e1767-e8.
48. Vera-Lastra O, Ordinola Navarro A, Cruz Domiguez MP, Medina G, Sánchez Valadez TI, Jara LJ. Two Cases of Graves' Disease Following SARS-CoV-2 Vaccination: An Autoimmune/Inflammatory

Syndrome Induced by Adjuvants. Thyroid. 2021;31(9):1436-9.
49. Lui DTW, Lee KK, Lee CH, Lee ACH, Hung IFN, Tan KCB. Development of Graves' Disease After SARS-CoV-2 mRNA Vaccination: A Case Report and Literature Review. Front Public Health. 2021;9:778964.
50. Weintraub MA, Ameer B, Sinha Gregory N. Graves Disease Following the SARS-CoV-2 Vaccine: Case Series. J Investig Med High Impact Case Rep. 2021;9:23247096211063356.
51. Sriphrapradang C, Shantavasinkul PC. Graves' disease following SARS-CoV-2 vaccination. Endocrine. 2021;74(3):473-4.
52. Pierman G, Delgrange E, Jonas C. Recurrence of Graves' Disease (a Th1-type Cytokine Disease) Following SARS-CoV-2 mRNA Vaccine Administration: A Simple Coincidence? Eur J Case Rep Intern Med. 2021;8(9):002807.
53. Patrizio A, Ferrari SM, Antonelli A, Fallahi P. A case of Graves' disease and type 1 diabetes mellitus following SARS-CoV-2 vaccination. J Autoimmun. 2021;125:102738.
54. Goblirsch TJ, Paulson AE, Tashko G, Mekonnen AJ. Graves' disease following administration of second dose of SARS-CoV-2 vaccine. BMJ Case Rep. 2021;14(12).
55. Sriphrapradang C. Aggravation of hyperthyroidism after heterologous prime-boost immunization with inactivated and adenovirus-vectored SARS-CoV-2 vaccine in a patient with Graves' disease. Endocrine. 2021;74(2):226-7.
56. Rubinstein TJ. Thyroid Eye Disease Following COVID-19 Vaccine in a Patient With a History Graves' Disease: A Case Report. Ophthalmic Plast Reconstr Surg. 2021;37(6):e221-e3.
57. di Filippo L, Castellino L, Giustina A. Occurrence and response to treatment of Graves' disease after COVID vaccination in two male patients. Endocrine. 2022;75(1):19-21.
58. Taieb A, Sawsen N, Asma BA, Ghada S, Hamza E, Yosra H, et al. A rare case of grave's disease after SARS-CoV-2 vaccine: is it an adjuvant effect? Eur Rev Med Pharmacol Sci. 2022;26(7):2627-30.
59. Chee YJ, Liew H, Hoi WH, Lee Y, Lim B, Chin HX, et al. SARS-CoV-2 mRNA Vaccination and Graves' Disease: a report of 12 cases and review of the literature. J Clin Endocrinol Metab. 2022.
60. Patrizio A, Ferrari SM, Antonelli A, Fallahi P. Worsening of Graves' ophthalmopathy after SARS-CoV-2 mRNA vaccination. Autoimmun Rev. 2022:103096.
61. Park KS, Fung SE, Ting M, Ozzello DJ, Yoon JS, Liu CY, et al. Thyroid eye disease reactivation associated with COVID-19 vaccination. Taiwan J Ophthalmol. 2022;12(1):93-6.
62. Hamouche W, El Soufi Y, Alzaraq S, Okafor BV, Zhang F, Paras C. A case report of new onset graves' disease induced by SARS-CoV-2 infection or vaccine? J Clin Transl Endocrinol Case Rep. 2022;23:100104.
63. Bostan H, Ucan B, Kizilgul M, Calapkulu M, Hepsen S, Gul U, et al. Relapsed and newly diagnosed Graves' disease due to immunization against COVID-19: A case series and review of the literature. J Autoimmun. 2022;128:102809.
64. Singh G, Howland T. Graves' Disease Following COVID-19 Vaccination. Cureus. 2022;14(4):e24418.
65. Chua MWJ. Graves' disease after COVID-19 vaccination. Ann Acad Med Singap. 2022;51(2):127-8.
66. Manta R, Martin C, Muls V, Poppe KG. New-onset Graves' disease following SARS-CoV-2 vaccination: a case report. Eur Thyroid J. 2022;11(4).
67. Sakai M, Takao K, Kato T, Ito K, Kubota S, Hirose T, et al. Graves' Disease after Administration of Severe Acute Respiratory Syndrome Coronavirus 2 (SARS-CoV-2) Vaccine in a Type 1 Diabetes Patient. Intern Med. 2022;61(10):1561-5.
68. Cuenca D, Aguilar-Soto M, Mercado M. A Case of Graves' Disease Following Vaccination with the Oxford-AstraZeneca SARS-CoV-2 Vaccine: Case Report and Review of the Literature. Eur J Case Rep Intern Med. 2022;9(4):003275.
69. Ruggeri RM, Giovanellla L, Campennì A. SARS-CoV-2 vaccine may trigger thyroid autoimmunity: real-life experience and review of the literature. J Endocrinol Invest. 2022.
70. Giusti M, Maio A. Acute thyroid swelling with severe hypothyroid myxoedema after COVID-19 vaccination. Clin Case Rep. 2021;9(12):e05217.
71. Capezzone M, Tosti-Balducci M, Morabito EM, Caldarelli GP, Sagnella A, Cantara S, et al. Silent thyroiditis following vaccination against COVID-19: report of two cases. J Endocrinol Invest. 2022;45(5):1079-83.
72. Nakaizumi N, Fukata S, Akamizu T. Painless thyroiditis following mRNA vaccination for COVID-

19. Hormones (Athens). 2022.
73. Yamamoto K, Mashiba T, Takano K, Suzuki T, Kami M, Takita M, et al. A Case of Exacerbation of Subclinical Hyperthyroidism after First Administration of BNT162b2 mRNA COVID-19 Vaccine. Vaccines (Basel). 2021;9(10).
74. Marsukjai A, Theerasuwipakorn N, Tumkosit M, Chattranukulchai P, Srichomkwun P, Prechawat S. Concomitant myocarditis and painless thyroiditis after AstraZeneca coronavirus disease 2019 vaccination: a case report. J Med Case Rep. 2022;16(1):212.
75. Poli K, Poli S, Ziemann U. Multiple Autoimmune Syndromes Including Acute Disseminated Encephalomyelitis, Myasthenia Gravis, and Thyroiditis Following Messenger Ribonucleic Acid-Based COVID-19 Vaccination: A Case Report. Front Neurol. 2022;13:913515.
76. Taylor P, Allen L, Shrikrishnapalasuriyar N, Stechman M, Rees A. Vaccine-induced thrombosis and thrombocytopenia with bilateral adrenal haemorrhage. Clin Endocrinol (Oxf). 2021.
77. Varona JF, García-Isidro M, Moeinvaziri M, Ramos-López M, Fernández-Domínguez M. Primary adrenal insufficiency associated with Oxford-AstraZeneca ChAdOx1 nCoV-19 vaccine-induced immune thrombotic thrombocytopenia (VITT). Eur J Intern Med. 2021;91:90-2.
78. Tews HC, Driendl SM, Kandulski M, Buechler C, Heiss P, Stöckert P, et al. SARS-CoV-2 Vaccine-Induced Immune Thrombotic Thrombocytopenia with Venous Thrombosis, Pulmonary Embolism, and Adrenal Haemorrhage: A Case Report with Literature Review. Vaccines (Basel). 2022;10(4).
79. Blauenfeldt RA, Kristensen SR, Ernstsen SL, Kristensen CCH, Simonsen CZ, Hvas AM. Thrombocytopenia with acute ischemic stroke and bleeding in a patient newly vaccinated with an adenoviral vector-based COVID-19 vaccine. J Thromb Haemost. 2021;19(7):1771-5.
80. D'Agostino V, Caranci F, Negro A, Piscitelli V, Tuccillo B, Fasano F, et al. A Rare Case of Cerebral Venous Thrombosis and Disseminated Intravascular Coagulation Temporally Associated to the COVID-19 Vaccine Administration. J Pers Med. 2021;11(4).
81. Maguire D, McLaren DS, Rasool I, Shah PM, Lynch J, Murray RD. ChAdOx1 SARS-CoV-2 vaccination: A putative precipitant of adrenal crises. Clin Endocrinol (Oxf). 2021.
82. Haji N, Ali S, Wahashi EA, Khalid M, Ramamurthi K. Johnson and Johnson COVID-19 Vaccination Triggering Pheochromocytoma Multisystem Crisis. Cureus. 2021;13(9):e18196.
83. Al Rawahi B, BaTaher H, Jaffer Z, Al-Balushi A, Al-Mazrouqi A, Al-Balushi N. Vaccine-induced immune thrombotic thrombocytopenia following AstraZeneca (ChAdOx1 nCOV19) vaccine-A case report. Res Pract Thromb Haemost. 2021;5(6):e12578.
84. Graf, Anneke, Armeni, Eleni, Dickinson, Louise, et al. Adrenal haemorrhage and infarction in the setting of vaccine-induced immune thrombocytopenia and thrombosis after SARS-CoV-2 (Oxford–AstraZeneca) vaccination2022; (1).
85. Efthymiadis A, Khan D, Pavord S, Pal A. A case of ChAdOx1 vaccine-induced thrombocytopenia and thrombosis syndrome leading to bilateral adrenal haemorrhage and adrenal insufficiency. Endocrinol Diabetes Metab Case Rep. 2022;2022.
86. Markovic N, Faizan A, Boradia C, Nambi S. Adrenal Crisis Secondary to COVID-19 Vaccination in a Patient With Hypopituitarism. AACE Clin Case Rep. 2022;8(4):171-3.
87. Murvelashvili N, Tessnow A. A Case of Hypophysitis Following Immunization With the mRNA-1273 SARS-CoV-2 Vaccine. J Investig Med High Impact Case Rep. 2021;9:23247096211043386.
88. Lindner G, Ryser B. The syndrome of inappropriate antidiuresis after vaccination against COVID-19: case report. BMC Infect Dis. 2021;21(1):1000.
89. Piñar-Gutiérrez A, Remón-Ruiz P, Soto-Moreno A. Case report: Pituitary apoplexy after COVID-19 vaccination. Med Clin (Barc). 2021.
90. Roncati L, Manenti A. Pituitary apoplexy following adenoviral vector-based COVID-19 vaccination. Brain Hemorrhages. 2022.
91. Bouça B, Roldão M, Bogalho P, Cerqueira L, Silva-Nunes J. Central Diabetes Insipidus Following Immunization With BNT162b2 mRNA COVID-19 Vaccine: A Case Report. Front Endocrinol (Lausanne). 2022;13:889074.
92. Ankiredypalli A, Chow LS, Radulescu A, Kawakami Y, Araki T. A case of hypophysitis associated with SARS-CoV2 vaccination. AACE Clin Case Rep. 2022.
93. Morita S, Tsuji T, Kishimoto S, Uraki S, Takeshima K, Iwakura H, et al. Isolated ACTH deficiency following immunization with the BNT162b2 SARS-CoV-2 vaccine: a case report. BMC Endocr Disord. 2022;22(1):185.

94. Mizuno T, Takahashi R, Kamiyama T, Suzuki A, Suzuki M. Neuroleptic Malignant Syndrome with Adrenal Insufficiency After BNT162b2 COVID-19 Vaccination in a Man Taking Valproate: A Case Report. Am J Case Rep. 2022;23:e936217.
95. Ohuchi K, Amagai R, Tamabuchi E, Kambayashi Y, Fujimura T. Fulminant type 1 diabetes mellitus triggered by coronavirus disease 2019 vaccination in an advanced melanoma patient given adjuvant nivolumab therapy. J Dermatol. 2022;49(5):e167-e8.
96. Yano M, Morioka T, Natsuki Y, Sasaki K, Kakutani Y, Ochi A, et al. New-onset Type 1 Diabetes after COVID-19 mRNA Vaccination. Intern Med. 2022;61(8):1197-200.
97. Tang X, He B, Liu Z, Zhou Z, Li X. Fulminant type 1 diabetes after COVID-19 vaccination. Diabetes Metab. 2022;48(2):101324.
98. Sasaki K, Morioka T, Okada N, Natsuki Y, Kakutani Y, Ochi A, et al. New-onset fulminant type 1 diabetes after severe acute respiratory syndrome coronavirus 2 vaccination: A case report. J Diabetes Investig. 2022.
99. Sasaki H, Itoh A, Watanabe Y, Nakajima Y, Saisho Y, Irie J, et al. Newly developed type 1 diabetes after coronavirus disease 2019 vaccination: A case report. J Diabetes Investig. 2022.
100. Sakurai K, Narita D, Saito N, Ueno T, Sato R, Niitsuma S, et al. Type 1 diabetes mellitus following COVID-19 RNA-based vaccine. J Diabetes Investig. 2022.
101. Makiguchi T, Fukushima T, Tanaka H, Taima K, Takayasu S, Tasaka S. Diabetic ketoacidosis shortly after COVID-19 vaccination in a non-small-cell lung cancer patient receiving combination of PD-1 and CTLA-4 inhibitors: A case report. Thorac Cancer. 2022;13(8):1220-3.
102. Bleve E, Venditti V, Lenzi A, Morano S, Filardi T. COVID-19 vaccine and autoimmune diabetes in adults: report of two cases. J Endocrinol Invest. 2022;45(6):1269-70.
103. Aydoğan B, Ünlütürk U, Cesur M. Type 1 diabetes mellitus following SARS-CoV-2 mRNA vaccination. Endocrine. 2022.
104. Sato T, Kodama S, Kaneko K, Imai J, Katagiri H. Type 1 Diabetes Mellitus Associated with Nivolumab after Second SARS-CoV-2 Vaccination, Japan. Emerg Infect Dis. 2022;28(7):1518-20.
105. Yakou F, Saburi M, Hirose A, Akaoka H, Hirota Y, Kobayashi T, et al. A Case Series of Ketoacidosis After Coronavirus Disease 2019 Vaccination in Patients With Type 1 Diabetes. Front Endocrinol (Lausanne). 2022;13:840580.
106. Ganakumar V, Jethwani P, Roy A, Shukla R, Mittal M, Garg MK. Diabetic ketoacidosis (DKA) in type 1 diabetes mellitus (T1DM) temporally related to COVID-19 vaccination. Diabetes Metab Syndr. 2022;16(1):102371.
107. Zilbermint M, Demidowich AP. Severe Diabetic Ketoacidosis After the Second Dose of mRNA-1273 COVID-19 Vaccine. J Diabetes Sci Technol. 2022;16(1):248-9.
108. Infante M, Fabbri A, Padilla N, Pacifici F, Di Perna P, Vitiello L, et al. BNT162b2 mRNA COVID-19 Vaccine Does Not Impact the Honeymoon Phase in Type 1 Diabetes: A Case Report. Vaccines (Basel). 2022;10(7).
109. Chatzimeletiou K, Fleva A, Sioga A, Georgiou I, Nikolopoulos TT, Markopoulou M, et al. Effects of Different Drug Therapies and COVID-19 mRNA Vaccination on Semen Quality in a Man with Ankylosing Spondylitis: A Case Report. Medicina (Kaunas). 2022;58(2).
