# Supplementary material for: Endocrine system after 2 years of COVID-19 vaccines: A narrative review of the literature
Source: Front Endocrinol (Lausanne). 2022 Nov 10;13:1027047. doi: 10.3389/fendo.2022.1027047 (PMC9685624; doi:10.3389/fendo.2022.1027047)
Supplement: Supplementary file 2 [file Table_2.pdf]

Supplementary Table 2. **Clinical studies concerning thyroid gland, type 1 diabetes mellitus and reproductive function after COVID-19 vaccine.**

Thyroid gland

Author, year	Study design	Patient(s)	Type of vaccine	Endpoint	Time between vaccination and assessment	Main outcome/Conclusions	Limitations
**Garcia et al, 2022 (20)**	Case/non-case study	162 cases of SAT among 1,221,582 spontaneous cases of adverse reactions with the four vaccines	Pfizer/BioNtecBNT162b2 (103), Moderna mRNA-1273 (27), Vaxzevria ChAdOx1-S (31) and Janssen Ad26.COV2.S (1)	To assess the association between SAT and COVID-19 vaccines, calculating the reporting odds ratios	Median time to onset of SAT (after any vaccine): 10 days	SAT was relatively more frequently reported in association with mRNA COVID-19 vaccines than with other viral vaccines.	Underreporting, overreporting and reporting bias; multiple confounding factors (comorbidity); the absence of critical data hinders a proper causality assessment for some of the cases
**Bostan et al, 2022 (21)**	Retrospective study	55 patients diagnosed with SATm, of whom 16 had undergone vaccination against COVID-19	Pfizer/BioNtecBNT162b2 (6), Sinovac Biotech CoronaVac in 6 (10)	To identify cases of vaccine–associated SAT, analyse the characteristics of these cases, and compare them with cases of non-vaccine associated SAT diagnosed in the same period	Median time to onset of symptoms after vaccination was 6.5 days	No differences between vaccinated and unvaccinated SAT patients in terms of age, gender, time to diagnosis, thyroid volumes, thyroid function tests, and acute phase reactants. No differences concerning treatment (methylprednisolone), follow-up duration and the frequency of euthyroidism at the follow-up visit	Retrospective design; limited sample size; lack of long-term follow-up
**Topaloglu et al, 2022 (22)**	Observational, retrospective cohort study	23 patients with vaccine related SAT and 62 patients with classical SAT	Pfizer/BioNtecBNT162b2 (n = 18), Sinovac Biotech CoronaVac in 6 (n = 5)	To investigate the differences in clinical, demographic, radiological and laboratory parameters between the patients with classical SAT and those with SAT developed after SARS-CoV-2 vaccines	Median time between vaccine and SAT development was 45 days (7-90), similar for both vaccine types; mostly after 2nd dose	Similar clinical features between vaccine related SAT and classical SAT. However, SAT duration was longer in vaccinated patients and TSH elevation was more frequently observed	Single center study, small number of patients
**Sendur et al, 2022 (23)**	Case–control study	14 patients with SARS- CoV-2 vaccine-induced SAT and 100 healthy controls	Pfizer/BioNtecBNT162b2 in 8 patients (57%) and Sinovac	To assess possible association between specific HLA genotypes and	NA	The frequencies of HLA-B35 and HLA-C04 alleles were higher in vaccine induced SAT patients compared with controls. Homozygosity for HLA-B35 and	Total number of cases was low; lack of detailed information in the control group regarding COVID-19 status, vaccine status, and other risk factors related to SAT;

			Biotech CoronaVac in 6 (43%)	development of SAT after vaccine		HLA-C04 was associated with a greater inflammatory reaction	size of control group too small to detect differences in low frequency alleles
**Paschou et al,2022 (26)**	Prospective study	72 healthy subjects (19 M, 53 W) with no history of thyroid disease	Pfizer/BioNtech BNT 16b2	To identify alteration of thyroid hormones and thyroid auto-antibodies after vaccination	50 days after the 2nd dose	Despite TSH and fT3 decrease, all thyroid hormone levels remained within the normal range. No changes were found for TPOAb or TGAb	Relatively small sample size
**Lui et al, 2022 (39)**	Prospective study	215 subjects with pre-vaccinal normal thyroid function	Pfizer/BioNtecBNT162b2 (129), Sinovac Biotech CoronaVac in 6 (86)	To evaluate the impact of COVID-19 vaccination on thyroid function and antibodies	First exams performed before vaccination; second exams performed after 2 months (about one month after 2nd dose)	After vaccination, TSH did not change, with slight increase of fT4 and slight decrease of fT3, within normal range. Only 3 patients had abnormal thyroid function (subclinical). TPOAb and TgAb slightly increased, without significant changes (TPOAb titre was greater in patients receiving Pfizer/BioNtecBNT162b2 vaccine)	Short follow-up; regarding only mRNA vaccines
**Li et al, 2022 (40)**	Prospective study	657 patients receiving inactivated vaccine, of which 564 had normal thyroid function and 545 had negative anti-thyroid antibodies	Sinopharm Beijing or Sinovac Biotech CoronaVac	To determine the potential impact of SARS-CoV-2 vaccines on the thyroid.	Within the first 28 days after the second dose	Among the patients with normal thyroid function at baseline, 36 (6.38%) developed thyroid dysfunction. None developed abnormal antibodies after vaccination.	Short follow-up period
**Xiong et al, 2022 (41)**	Retrospective cohort study	47,086 patients under levothyroxine of which 23423 unvaccinated	Pfizer/BioNtech BNT 16b2 (12310 patients); Sinovac Biotech Coronavac (11353 patients)	To evaluate the risks of adverse events after COVID-19 vaccination among patients treated for hypothyroidism	After 2nd dose in > 95% of cases (no information on specific timing)	BNT162b2 or CoronaVac vaccination were not associated with alterations of thyroid status or an increased risk of adverse outcomes among patients under treatment for hypothyroidism	Details about the aetiologies of hypothyroidism were not available; Levothyroxine dosage was used as a surrogate of thyroid status; Drug adherence could not be ascertained; Retrospective nature

Glycemic alterations in T1DM Patients

**Author, year**	**Study design**	**Patient(s)**	**Type of vaccine**	**Endpoint**	**Time between vaccination and assessment**	**Main outcome/Conclusions**	**Limitations**

**Heald et al, 2021 (76)**	Retrospecti ve study	97 (51W, 46 M) with T1DM, median age 44 years, median HbA1c 7.6%	Pfizer/BioNtech BNT 16b2 (45) Vaxzevria ChAdOx1 (52)	To assess instability of blood glucose levels after COVID-19 vaccination.	7 days after 1st dose (compared to 7 days before vaccine)	Vaccination can cause temporary perturbation of interstitial glucose (58% showed a reduction in time in target range), with this effect more pronounced in people talking oral hypoglycaemic medication plus insulin, and when HbA1c is lower.<br><br>No differences between the vaccine type.	Not quantified what changes were made in the insulin doses after vaccine<br><br>No measurement of inflammatory markers<br><br>Not compared first and second dose of vaccine effects on interstitial glucose regulation
**Haeld et al, 2021 (77)**	Retrospecti ve study	20 (11W, 9M) with T1DM, median age 53 years, median HbA1c 7.3%	Pfizer/BioNtech BNT 16b2 (8), Vaxzevria ChAdOx1 (12)	To assess instability of blood glucose levels after COVID-19 vaccination.	7 days after 1st dose (compared to 7 days before vaccination)	Temporary (1 week) hyperglycaemia especially in patients taking insulin + oral hypoglycaemics and in older individuals. No differences between the vaccine type.	Not quantified what (if any) changes were made in the insulin doses during the week following the COVID-19 vaccine.
**Aberer et al, 2022 (78)**	Multicenter prospective study	58 with T1DM, mean age 39.5 ± 14.1, mean HbA1c 57 ± 12 mol/mol	Pfizer/BioNtech BNT 16b2 or Moderna mRNA-1273 or Vaxzevria ChAdOx1	Time spent in different glycemic ranges	From 2 days prior until 3 days after the 1st dose	No significant differences were found for the TIR	Low sample size
**D'onofrio et al, 2021 (79)**	Retrospecti ve study	35 (14W, 21M) with T1DM, median age 36 years, median HbA1c 7.6%,	Pfizer/BioNtech BNT 16b2	Differences in TIR after the 1st and 2nd dose of vaccine compared to TIR before the 1st and 2nd dose of vaccine	3 days (compared to 14 days before vaccination)	No significant differences in TIR between before and after the 1st and 2nd vaccination dose	Low sample size and by the single-centre designs<br><br>Young age<br><br>Short duration of the follow-up
**Piccini et al, 2022 (80)**	Retrospecti ve study	39 (17W, 22M) with T1DM, mean age 18.7 ± 2.1	Pfizer/BioNtech BNT 16b2<br><br>or<br><br>Moderna mRNA-1273	To evaluate glycaemic control modification, insulin dose adjustment and adverse effects after COVID-19 vaccination in young T1DM individuals	7 and 14 days before and following the 1st and 2nd dose of the vaccine	No significant differences in TIR were observed before and after any dose nor before and after the whole vaccination cycle	Low sample size and the single-centre design

**Dicembrini et al, 2022 (81)**	Observational retrospective study	454 with T1DM (205W, 249M), median age 52.5 years, mean HbA1c of 7.4%.	Moderna mRNA-1273 SARS-CoV-2 vaccine	Effects of vaccine on glucose variability	7 days after 1st and 2nd dose (compared to 7 days before vaccination)	No significant difference was observed in mean glucose, glucose coefficient of variation, time in range, time in hyperglycaemia or time in hypoglycaemia	

Female reproductive system

Author, year	Study design	Patients	Type of vaccine	Endpoint	Time between vaccination and assessment	Main outcome/Conclusions	Limitations
**A. Mohr-Sasson et al., 2022 (82)**	Prospective study	132 W (18–42 years; mean age 29 years)	Pfizer/BioNtech BNT 16b2	To evaluate the association between vaccine and ovarian reserve in terms of circulating AMH levels	AMH plasma levels were collected at recruitment and three months after the first vaccination	Difference between AMH levels before and after the vaccine was not statistically significant.	No randomized unvaccinated control group.<br>Short period of follow-up.
**Bentov et al, 2021 (83)**	Cohort study	32 W, IVF patients of which 9 vaccinated, 7 with previous covid infection and 16 not vaccinated/ infected; mean age 33.7 years	Pfizer/BioNtech BNT 16b2	To evaluate if the immune response to SARS-CoV-2 infection or Pfizer-BioNTech vaccination affects human follicular function	Mean time periods from recovery to sampling is 98.14 days (range 48-169 days)	Neither infection or vaccine nor the immune response to them resulted in any measurable detrimental effect on the function of the ovarian follicle	Small sample size and presence of fertile and infertile population.<br>Non-exclusion of later sequelae.
**Muhaidat et al., 2022 (84)**	Cross-sectional study	2269 W (mean age 34.3 ± 8.5 years)	Any type of vaccine	To investigate the prevalence and impact of menstrual abnormalities after COVID-19 vaccine	Online self-administered survey from July 2021 to August 2021	66.3% of participants reported menstrual symptoms post-vaccination, of which 46.7% after their first dose. In 93.6% of participants, the symptoms resolved within 2 months. Vaccine type did not significantly influence the incidence of abnormalities	Cross-sectional design<br>Self-reported data extraction via internet-based survey
**Edelman et al, 2022 (85)**	Retrospective cohort study	2,403 W vaccinated vs 1,556 W unvaccinated, aged 18-45 years	Pfizer/BioNtech BNT 16b2 (55%) Moderna mRNA-1273 (35%), Janssen Ad26.COV2.S (7%)	To evaluate whether patients who have received vaccine reported changes in menses length vs unvaccinated patients.	Six consecutive cycles: three pre vaccine cycles and three post-first vaccine dose	The vaccinated cohort experienced a 0.64 days increase in the length of their menstrual cycle during the first vaccine cycle compared with their three prevaccination cycles; the unvaccinated cohort had no significant change in cycle four compared with their first three	Sample collected by the users of “Natural Cycle”.<br>Cohort with consistent normal cycle lengths.<br>No data available on severe acute respiratory syndrome SARS-CoV-2 infection in either groups

						cycles. 10% of the subgroup of 358 women who received both vaccine doses within a single cycle had an increase in cycle length of 8 days or more	
**Laganà et al., 2022 (86)**	Cross-sectional study	164 W mean age 35.8 ± 7.2 years, with self-reported regular menses before vaccine	Any type of vaccine	To investigate menstrual irregularities after the first and second doses of the COVID-19 vaccine	Self-administered questionnaire from 10 September to 10 October 2021	After the first dose of vaccine, from 33.3% to 66.7% of women (according to vaccine type) reported cycle length alterations, mainly a shortening of menstrual cycle (1–5 days earlier). After the second dose of vaccine, from 52.5% to 78.6% (according to vaccine type) reported cycle length alterations, mainly a shortening of menstrual cycle (1–5 days earlier)	Number of women Self-administered questionnaire
**Lessans et al, 2022 (87)**	Questionnaire-based cross-sectional study	219 W, aged 18-50 without known gynaecological comorbidities and who regularly monitor their menstruation	Pfizer/BioNtech BNT 16b2	To investigate the impact of the BNT162b2 vaccine on women's menstural cycle	Three months before and after receiving the vaccine	Almost 40% of women experienced menstrual changes and almost one fifth reported irregular bleeding following the vaccination (of them, 66.7% reported irregular bleeding that preceded their estimated menstrual and 33.3% reported a delay). Nearly 68% of study participants reported dysmenorrhea following vaccination.	Retrospective nature; possible recall bias; possibility of personal interpretation of some of the questions; lack of a control group
**Lee et al, 2022 (88)**	Observational retrospective study	39,129 W, median age 33 years (18-80)	Pfizer/BioNtech BNT 16b2, Moderna mRNA-1273, Vaxzevria ChAdOx1, Janssen Ad26.COV2, Novavax, and other vaccines	To investigate whether the current SARS-CoV-2 vaccines affect menstrual cycling or menstruation	At least 14 days after completing vaccinal cycle (according to which vaccine was administered)	42% of people with regular menstrual cycles bled more heavily after vaccination, 44% reported no changes. Among women who did not typically menstruate, 71% of those on long-acting reversible contraceptives, 39% of those on gender-affirming hormones, and 66% of postmenopausal ones reported breakthrough bleeding	Possible selection bias

Male reproductive system

**Author, year**	**Study design**	**Patients**	**Type of vaccine**	**Endpoint**	**Time between vaccination and assessment**	**Main outcome/Conclusions**	**Limitations**

**Carto et al, 2021 (90)**	Retrospective comparative cohort study	663,774 M vaccinated vs 663,774 M not vaccinated, aged ≥ 12 years	Any type of vaccine (one or more dose)	To assess the risk of developing orchitis and/or epididymitis in a cohort of men not vaccinated vs vaccinated	Between 1 and 9 months after vaccination	Men who had received a COVID-19 vaccine were significantly less likely to develop orchitis and/or epididymitis: 340 patients in the vaccinated cohort were diagnosed with orchitis and/or epididymitis, while 8,257 patients in the unvaccinated cohort had orchitis and/or epididymitis in the same time window.	Retrospective cohort studies using claims databases.
**Gonzalez et al, 2021 (91)**	Prospective study	45 M, healthy volunteers aged 18 to 50 years	Pfizer/BioNtech BNT 16b2 or Moderna mRNA-1273	To assess sperm parameters before and after mRNA vaccine administration	Participants provided a semen sample prior to receiving the 1st dose and 70 days after the 2nd dose	There were no significant decreases in any sperm parameter. Men with oligospermia did not experience further decline after vaccination.	Small cohort of healthy men
**Reschini et al, 2022 (92)**	Multicentre retrospective study	106 M, median age 39 years, followed up at an infertility center	Pfizer/BioNtech BNT 16b2 or Moderna mRNA-1273 or Vaxzevria ChAdOx1 or Janssen Ad26.COV2.S	To assess semen parameters and fertilization rate in a pairwise comparison between the first and second assisted reproduction technology attempt, performed respectively before and after COVID-19 vaccination	Median time between the first vaccine dose and the second assisted reproduction technology cycle was 75 days	Fertilization rate was similar before and after vaccination. The various semen parameters did not change before and after the exposure. None of the patients was azoospermic after the vaccination nor one had a severe deterioration of the semen parameter. No difference was observed even after considering different types of vaccines (mRNA or viral vector).	/
**Barda et al, 2022 (93)**	Prospective observational cohort study	33 M, mean age 27 years, who were sperm donors at the sperm bank of a fertility center	Pfizer/BioNtech BNT 16b2	To examine the effect of the SARS-CoV-2 virus vaccine on sperm quality	All sperm donors donated sperm repeatedly before and 72 (or more) days after receiving the SARS-CoV-2 vaccine (two doses).	No deleterious effect on sperm quality for both fresh and frozen, thawed samples. Sperm quality improved after two doses of vaccine, as compared to sperm samples given in the months prior to vaccination. Sperm samples that were obtained after the first vaccine dose also did not show any negative effect on sperm quality, which was found to be improved compared to samples before vaccination.	The number of sperm samples available for analysis differed between subjects, according to the number of samples given during each period (before or after vaccination).
**Safrai et al, 2022 (94)**	Retrospective study	72 M, median age 35.7 years, undergoing in vitro	Pfizer/BioNtech BNT 16b2	To assess the effect of vaccine on sperm parameters of men with normal or	Records of the same patients were reviewed before and after vaccine. Median time between	Sperm parameters showed no significant changes after vaccination among men with a normal and abnormal semen analysis. Except sperm volume,	Retrospective data collection of the sperm parameters of the pre-vaccine group; relatively long interval between the pre- and post-vaccination semen analyses.

		fertilization treatment.		impaired sperm parameters.	the first vaccine and the post-vaccine sperm analysis was 71 days.	which had a slightly decrease, none of the parameters differed after vaccination.	
**Lifshitz et al, 2022 (95)**	Prospective cohort study	75 M, fertile (with a child without the use of artificial reproductive technology) and younger than 45 years	Pfizer/BioNtech BNT 16b2	To assess the percentage of abnormal semen parameters in men who were vaccinated	Semen samples were analysed 1–2 months following the second dose of vaccine	The semen parameters following COVID-19 vaccination were predominantly within the normal reference ranges.	Men were only tested once after they were vaccinated and not before vaccination.
**Olana et al, 2022 (96)**	Single-center prospective study	47 M (age: 29.3 ± 6.0 years, BMI: 23.15 ± 2.5 kg/m2)	Pfizer/BioNtech BNT 16b2	To compare spermatozoa parameters before and after vaccine inoculation (two doses, one month apart)	Semen samples were analysed before the first dose, and three months after the first dose	No significant differences were observed in semen parameters in T0, compared to T1 for volume, concentration, total number, total and progressive motility, abnormal forms (confirmed also in sub analysis group including only oligo and asthenozoospermic patients)	Men were selected from a specific population; small number of oligo-zoospermic individuals
**Gat et al, 2022 (97)**	Retrospective multicenter study	37 M (sperm donors) who supplied at least single semen sam-ple prior and post vaccination	Pfizer/BioNtech BNT 16b2	To investigate the effect of covid-19 vaccine on semen parameters among semen donors	Semen samples were analysed before and after an average of 26.7, 92.5, and 174.8 days post-vaccination	No significant change was demonstrated between pre-vaccine and the first control post-vaccine. Sperm concentration and motility were significantly lower at second control post vaccine compared to pre-vaccine data, but with a recovery at third control post vaccine	The study focused on sperm donors rather than the general population; retrospective design
**Xia et al, 2022 (98)**	Retrospective cohort study	105 M vaccinated, 155 M unvaccinated (controls)	Sinovac Biotech Coronavac (70 men) Sinopharm Beijing (35 men)	To compare the sperm parameters, embryonic development and blastocysts quality between vaccinated and unvaccinated men in IVF	Not specified; to be included in the vaccinated group, patients had to have their vaccinal cycle completed	The sperm parameters between vaccinated and unvaccinated men were similar in terms of volume, sperm concentration, sperm count, total and progressive motility. No statistically difference was found in embryo quality of IVF	The number of participants is small; lack of data on newborns; no semen examination before vaccination
**Zhu et al, 2022 (99)**	Retrospective cohort study	43 M (sperm donors)	Inactivated COVID-19 Vaccines (2 doses)	To evaluate semen quality before and after receiving inactivated COVID-19 vaccine	1st donation within one month before the first dose; 2nd donation within 21 days after the first dose; and (3) donation within 60	No statistically significant changes in volume, sperm concentration, progressive motility and total progressive motile count	The sample size was limited, and all the participants' data were collected in a single center

					days after the second dose		
**Abd et al, 2022 (100)**	Two-centre prospective observation al study	60 M (age < 50 years), belonging to IVF couples experiencing infertility due to female factors	Pfizer/BioNtech BNT 16b2	To evaluate safety of the Pfizer-BioNTech vaccine on male fertility as represented by the semen parameters	1st sample before vaccine; 2nd sample at least 90 days after the second dose	Vaccination did not afect semen volume, pH, sperm concentration, or morphology. Nevertheless, there were statistically significant differences in total and progressive sperm motility	The sample size was limited

Couple fertility

**Author, year**	**Study design**	**Patients**	**Type of vaccine**	**Endpoint**	**Time between vaccination and assessment**	**Main outcome/Conclusions**	**Limitations**
**Orvieto et al, 2021 (101)**	Observatio nal study	36 couples (median age 37.3 W and 40.1 M) undergoing IVF treatment	Pfizer/BioNtech BNT 16b2	To assess the influence of vaccine on IVF treatments	Couples performed consecutive ovarian stimulation cycles for IVF before and 7-85 days after receiving mRNA SARS-CoV-2 vaccine (two doses)	No influence on couple fertility rate during their immediate subsequent IVF cycle: no detrimental effects of the vaccine on ovarian reserve and on developing gametes/embryos, with an acceptable pregnancy rate (30 % per transfer). No differences in ovarian stimulation and embryological variables before and after receiving vaccination.	Small sample size and short period of follow-up
**Odeh-Natour et al, 2022 (102)**	Prospective observation al cohort study	59 W undergoing IVF cycles; 37 vaccinated vs 22 not vaccinated	Pfizer/BioNtech BNT 16b2	To evaluate the effect of vaccine on IVF treatment, oocyte and embryo quality, and pregnancy outcome	Follicular fluid assessed 2-8 weeks after the second vaccination	No differences between patients who were vaccinated, infected or neither: IVF treatment and pregnancy outcomes were comparable between groups.	Small sample size and short period of follow-up
**Avraham et al, 2022 (103)**	Retrospecti ve age-matched cohort study	200 vaccinated W and 200 age-matched unvaccinated W	Pfizer/BioNtech BNT 16b2	To assess the influence of coronavirus vaccine on ovarian response and IVF treatment outcomes	Oocyte retrieval 14–68 days after second dose	No difference in the mean number of oocytes retrieved per cycle; no difference in the clinical pregnancy rates; the fertilization rates and mean number of cryopreserved embryos were similar	Retrospective nature; different treatment protocols used; lack of information about vaccination or past infection status of the male partners

**Wu et al, 2022 (104)**	Retrospective cohort study	240 vaccinated W and 1343 unvaccinated W	Sinovac Biotech Coronavac or Sinopharm	To investigate the effect of inactivated vaccines on IVF outcomes, especially ongoing pregnancies	91.7% of vaccinated women had received the second dose, 6.7% the first dose and 1.7% had completed the full course of vaccinations before stimulation	Vaccination before ovarian stimulation did not have any effects nor on IVF outcomes (rates of ongoing pregnancy, clinical pregnancy and early pregnancy loss) or on the number of oocytes retrieved and the development of the embryos (fertilization rate, blastocyst development rate, number of blastocysts and embryos suitable for transfer)	Retrospective nature; limited follow-up period (no live birth outcomes available)
**Wesselink et al, 2022 (105)**	Prospective cohort study	2126 W aged 21–45 years, belonging to couples trying to conceive spontaneously	Any type of vaccine	To examine the associations of fecundability, the per-cycle probability of conception, with COVID-19 vaccination and SARS-CoV-2 infection	73% of women and 74% of men had received at least 1 dose of COVID-19 vaccine by the final observed cycle (Questionnaires every 8 weeks for up to 12 months)	COVID-19 vaccination was not appreciably associated with fecundability in either partner. Female SARS-CoV-2 infection was not strongly associated with fecundability. Male infection was associated with a transient reduction in fecundability.	Internet enrollment

Abbreviations: TPOAb: thyroid peroxidase antibodies; TGAb: thyroglobulin antibodies; T1DM: type 1 diabetes mellitus; HbA1c: Glycosylated Hemoglobin, Type A1C ; TIR: time in range; AMH: anti-Müllerian hormone; IVF: in vitro fertilization
